# Systemic Administration of Abeta mAb Reduces Retinal Deposition of Abeta and Activated Complement C3 in Age-Related Macular Degeneration Mouse Model

**DOI:** 10.1371/journal.pone.0065518

**Published:** 2013-06-14

**Authors:** Ian Catchpole, Volker Germaschewski, Jaimie Hoh Kam, Peter Lundh von Leithner, Susannah Ford, Gerald Gough, Peter Adamson, Philip Overend, Jan Hilpert, Francisco J. López, Yin Shan Eric Ng, Pete Coffey, Glen Jeffery

**Affiliations:** 1 Topical BioPharm Discovery Research and Development Unit, King of Prussia, Philadelphia, Pennsylvania, United States of America; 2 Institute of Ophthalmology, University College London, London, United Kingdom; 3 GSK Ophthalmology, King of Prussia, Philadelphia, Pennsylvania, United States of America; 4 BioPharm Discovery Medicine, GlaxoSmithKline, Stevenage, Herts, United Kingdom; University of Cologne, Germany

## Abstract

Age-related macular degeneration (AMD) is a leading cause of legal blindness in the Western world. There are effective treatments for the vascular complications of neo-vascular AMD, but no effective therapies are available for the dry/atrophic form of the disease. A previously described transgenic CFH-gene deficient mouse model, (*cfh−/−*), shows hallmarks of early AMD. The ocular phenotype has been further analysed to demonstrate amyloid beta (Aβ) rich basement membrane deposits associated with activated complement C3. *Cfh−/−* mice were treated systemically in both prophylactic and therapeutic regimes with an anti-Aβ monoclonal antibody (mAb), 6F6, to determine the effect on the *cfh−/−* retinal phenotype. Prophylactic treatment with 6F6 demonstrated a dose dependent reduction in the accumulation of both Aβ and activated C3 deposition. A similar reduction in the retinal endpoints could be seen after therapeutic treatment. Serum Aβ levels after systemic administration of 6F6 show accumulation of Aβ in the periphery suggestive of a peripheral sink mechanism. In summary, anti-Aβ mAb treatment can partially prevent or reverse ocular phenotypes of the *cfh−/−* mouse. The data support this therapeutic approach in humans potentially modulating two key elements in the pathogenesis of AMD – Aβ and activated, complement C3.

## Introduction

Age-related macular degeneration (AMD) is the leading cause of blindness in those over 55 years in the developed world. There are two major clinical presentations of AMD. Atrophic (dry) AMD is characterised by the degeneration of retinal pigment epithelial (RPE) and the neural retina. Early stages of atrophic AMD are associated with the formation of drusen, under the RPE. Progression to an end stage disease, where the RPE degenerates completely and forms sharply demarcated areas of central cell loss, leading to regional loss of vision, is termed geographic atrophy. In a minority of AMD patients the disease progresses with the development of choroidal neovascularisation, (CNV), known as wet AMD, where the development of weak, leaky blood vessels can result in haemorrhage and complete blindness. There has been progress in developing treatments limiting blood vessel development and leakage with molecules which inhibit either vascular endothelial growth factor, (VEGF), or the VEGF receptor signalling pathway. However, there is neither treatment for the atrophic form of AMD nor for the prevention of its progression to wet AMD, [1].

There are no animal models which provide all the characteristics of human AMD pathology but there are some interesting findings and parallels shown in the ocular phenotypes of transgenic mice. Transgenic mice inactivated for the murine apolipoprotein E (*apoE*) gene, but expressing the human Apo E variant, *apoE3* Leiden, [2], and especially *apoE4*, show, on a high fat diet, ocular phenotypes ranging from basal laminar deposits under the RPE to drusen deposition and CNV [3]. Association of the *apoE* protein to the *apoE* receptor 2 has been shown to trigger the endocytosis of amyloid precursor protein, (APP) in neuroblastoma cells, leading to the production of amyloid beta (Aβ), [4]. Additionally, mice disrupted for the neprilysin gene, which encodes a peptidase that degrades Aβ, have increased deposition of Aβ under the RPE and also show increased RPE cell degeneration and a similar histology to that observed in human AMD. [5]. Mouse models of Alzheimer’s disease, (AD), over-expressing human APP, leading to brain and later retinal deposition of Aβ, suggest a role for Aβ in both AD and AMD, [6]–[9].

There are similarities between the drusen formation in AMD and in formation of plaques in AD. Drusen contain similar protein components to the plaques found in AD. ApoE and Aβ are found both in atrophic, AMD drusen and AD plaques. The Aβ found in drusen is thought to be locally derived from the RPE cells, [10]. The involvement of ageing and a secondary inflammatory process also appears to be common in both AMD and AD. In the AMD inflammatory process, there is an associated rise in expression of both Aβ protein and acute-proteins such as C-reactive protein (CRP). Both of these protein classes may induce both complement activation and the activation of pro-inflammatory cytokines. Activated complement components are also found in drusen and a number of polymorphisms in genes involved in the alternative complement pathway are associated with AMD development. Many polymorphisms have been described especially in the key regulator complement factor H, (CFH), but also in Factor B, C2 and C3. The implication of such polymorphisms is a dysfunctionally activated or regulated alternative complement pathway. Activated complement components lead to the formation of a final membrane attack complex which can lyse cells, releasing cytokines such as VEGF. In AD, deposition of plaques containing Aβ protein and neurofibrillary tangles are known to activate complement; thus linking disease mechanisms with AMD, [11]–[13].

The aged and aging complement factor H, (*cfh−/−)*, deficient mouse has a pathological retinal phenotype that may mirror some of the changes found in AMD, with elevated outer retinal deposits and complement C3 accumulation [14]. Here we investigate the effects of systemic administration of an anti-Aβ monoclonal antibody (mAb), in both prophylactic and therapeutic regimes, on the accumulation of Aβ and activated complement C3 in the retina of the *cfh−/−* mouse.

## Materials and Methods

### Ethics Statement

All animal studies were ethically reviewed and carried out in accordance with Animals (Scientific Procedures) Act 1986 (UK), the University College London ethics committee approval and the GSK Policy on the Care, Welfare and Treatment of Laboratory Animals under a UK Home Office project license (PPL 70/6571).

### Generation of Monoclonal Antibody, (mAb), 6F6, to Aβ

Mice were immunised, via the intra peritoneal route, with a synthetic peptide from a region of the human Aβ sequence: N-CGGGNKGAIIGLMVGG (27–38) conjugated to purified protein derivative, (PPD). Once the mice had reached optimal response titres, hybridomas were generated by obtaining the spleens cells and fusing these with myeloma cells, derived from X63/Ag8.653, [15], using PEG (polyethylene glycol) methodology. The resultant mixed cell population was then plated out into 96 well plates.

Hybridoma samples were screened against multiple forms of Aβ peptides including the immunogen used for immunization using Fluorometric Microvolume Assay Technology (FMAT). Further secondary screening was performed to confirm hits by on and off rate ranking of binding to N-terminal biotinylated Aβ (1–40) by Biacore and absence of binding to human APP expressed on HEK cells, (Biotechnology Research Institute, National Research Council, Canada). Epitope mapping by ELISA using a set of 31, 12-mer overlapping peptides (data not shown) which covered the complete sequence of the Aβ 1–42 peptide was carried out on selected hits. Antibody was purified on a large scale directly from the hybridoma. In depth examination of the binding kinetics with purified recombinant antibody material resulted in selection of IgG2a mAb 6F6. 6F6 showed low single digit nM affinity by Biacore to both human and rodent Aβ 1–40 and 1–42 (data not shown).

### Generation of mAb 5G5 and an IgG2A isotype-specific Control

5G5 is a GSK mouse monoclonal antibody specific for the C-terminus of Aβ 1–42, (epitope 35–42) and was generated in a similar manner to 6F6 by immunization of C57Bl/6 mice with human Aβ peptide 37–42 conjugated to KLH. In a similar manner to the generation of 6F6 and 5G5, an IgG2A mouse monoclonal antibody was generated against a peptide motive in the Human Papilloma Virus-11, E1 gene and this was used as an isotype-specific control in some experiments.

### Animals and Experimental Design

Mice, *cfh−/−* were backcrossed onto the C57BL/6 genetic background for more than 10 generations, were fed *ad libitum* and housed conventionally with a 12 hour day light/dark cycle. The anti-Aβ antibody 6F6 was given systemically to mice both prophylactically, (0.06, 0.3 & 0.6 mg), and therapeutically, (0.06 & 0.6mg), via intraperitoneal (IP) route, on a weekly dosing regimen. Time-points for respective treatment were determined based on the onset and progression of pathologies in the model. The prophylactic treatment was started at 3 months of age, before any significant accumulation of Aβ starts and the therapeutic treatment was started at the age of 6 months. The prophylactic treatment was continued for duration of 3 months and the therapeutic treatment was also performed for duration of 3 months with an intermediate time-point at 1 month.

### Prophylactic Treatment

Five groups of three-month old *cfh−/−* (±2 weeks), each containing five animals (n = 5) were used for the prophylactic treatment. The groups are described in detail in Table 1. To determine baseline levels for IHC analysis, five untreated, three-month old *cfh−/−* mice were culled and their eyes were removed and processed for immunostaining.

**Table 1:** Prophylactic administration schedule for 6F6 in *cfh−/−* mice.

Group	Description	Dose per animal (µg)	Dose (mg/kg)	Strain	Number/group
1	Vehicle (PBS)	–	–	*cfh−/−*	5
2	6F6	60	3	*cfh−/−*	5
3	6F6	300	15	*cfh−/−*	5
4	6F6	600	30	*cfh−/−*	5
5	Background levels ofexperimental endpoints in3 month old animals	–	–	*cfh−/−*	5

### Therapeutic Treatment

Four groups of six month old *cfh−/−* mice (±1 month), were used in the therapeutic treatment. The groups are described in detail in Table 2. A further group, (n = 4), was used as baseline and they were culled at 6 months of age and their eyes were removed and processed for immunohistochemistry. An additional group of wild type, (wt), C57BL/6 mice, (n = 4), aged six months were injected weekly with 6F6 at a 0.6 mg dose, to monitor for any effects of 6F6 on wt mice and these were culled and analyzed after one month (4 weeks) of treatment.

**Table 2:** Therapeutic administration schedule for 6F6 in *cfh−/−* mice.

Group	Description	Dose per animal (µg)	Dose (mg/kg)	Strain	Number/group per time-point (weeks)
1	Vehicle (PBS)	–	–	*cfh−/−*	4 (4 wks) 8 (12 wks)
2	6F6	60	3	*cfh−/−*	4 (4 wks) 8 (12 wks)
3	6F6	600	15	*cfh−/−*	4 (4 wks) 8 (12 wks)
4	Background levels ofexperimental endpointsin 3 month old animals	–	30	*cfh−/−*	4 (4 wks)
5	6F6 Background levelspre-treated & treated wild-type	600	–	C57Bl/6	4 (4 wks)

### Analysis of *cfh−/−* mice after Systemic Administration of 6F6

*Cfh−/−* mice were treated at three months, (prophylaxis), and at six months, (therapy), weekly for 12 weeks. Efficacy end points, for both regimes were IHC analysis and quantification of retinal deposition of Aβ and activated complement C3, demonstration of elevated levels of free Aβ (1–40 and 1–42) and total Aβ (1–42), free and mAb complexes in sera.

### *In vivo* Imaging

A modified confocal Scanning Laser Ophthalmoscope, (cSLO), (HRA2, Heidelberg, Germany) was used where the diameter of the confocal aperture was reduced to 100 µm and the power at the pupil of the 488 nm Argon laser increased to 1.4 mW to improve signal-to-noise. Reflectance images were made at 488 nm and 820 nm illumination and autofluorescence (AF) images were generated at 488 nm excitation and >500 nm emission. Tomographic image stacks were made of the retina, where the focal plane was sequentially moved vitreal to sclera to distinguish hyper-fluorescent points associated with the RPE from other fluorescing structures. All image sequences were captured at 8.9 Hz, 55° field-of-view, and were digitized as 8-bit, 1536×1536-pixel image files, resulting in an optimal lateral image resolution of 1.2 µm/pixel. Optimal axial resolution in the mouse eye was estimated to be 5–8 µm. The principal limiting factor affecting image quality and resolution was scattering due to clouding of cornea and/or lens cataract. Hyperfluorescent foci with a diameter >20 µm present within a 900 µm radius of the optic disk (OD) in *in vivo* confocal fluorescence fundus images made at the level of the subretinal space of both eyes in animals of each test group were quantified (retinae in eyes where excessive scattering in ocular media reduced resolution were excluded from analysis).

### Immunohistochemistry, (IHC)

**Figure 1:**
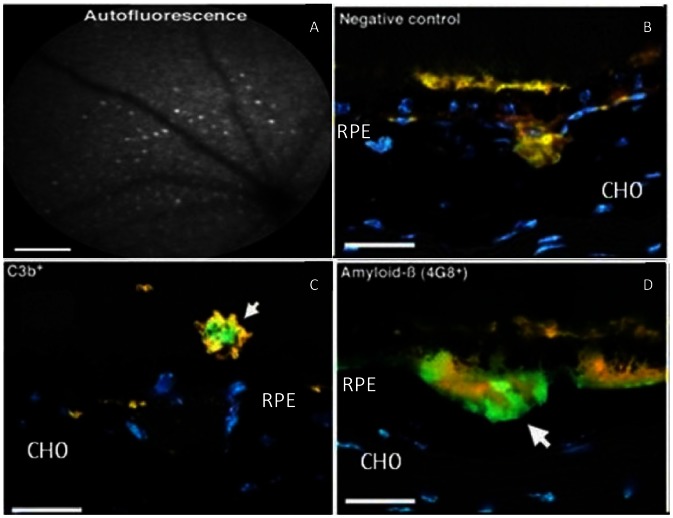
Retinal imaging and immunohistochemical analysis of 12 month old *cfh−/−* mice. (A) Scanning laser ophthalmoscope image of the retinae: Large clusters of hyperfluorescent foci (showing as autofluorescent point sources are observed. (B–D) Immunohistochemically labelled sections of the fluorescent debris/deposits, the majority of which may be containined within macrophages, often occur simultaneously on either side of the RPE. (B) acts as a negative control for Aβ detection by IHC (background signal from an isotype specific control antibody). The signal for fluorescent debris, (yellow), was dampened by use of Sudan Black in all cases. RPE-associated foci, show cross-reactivity, (green, highlighted with white arrows), to activated complement C3, (C3b+, detected with clone 2/11, #HM1065, Hycult Biotech, Table 3), (C), and Aβ, (4G8+), (D). A- scalebar = 200 µm; B–D scalebar = 25 µm. Blue label is DAPI, (4', 6-diamidino-2-phenylindole), a nuclear stain. CHO = choroid, RPE = retinal pigment epithelial cells.

Animals were euthanized by exposure to CO_2._ Both eyes of each animal were used. Eyes were removed and fixed in 4% paraformaldehyde in phosphate buffered saline (PBS), pH 7.4 for 1 h. After washing in PBS the eyes were cryoprotected in 30% sucrose in PBS, the lens removed and the eye cups frozen in OCT (Tissue Tek) using dry ice/acetone freezing slurry. Cryostat sections (10 µm) were thaw-mounted onto charged slides. IHC was performed at room temperature. On some occasions, (see data Figure 1), sections were pre-treated with Sudan Black in order to reduce autofluorescence signal from the retinal pigment epithelial cell/Bruch membrane interface. Retinal sections were blocked for 1 hr in 5% normal donkey serum in 0.1 M phosphate buffer saline (PBS), pH 7.4 with 0.3% Triton X-100, and incubated overnight with primary antibodies (see Table 3), diluted with 1% normal donkey serum in 0.1 M PBS with 0.3% Triton X-100. Primary antibody exposure was followed by washing, three times in 0.1 M PBS, and then incubated with respective secondary antibodies (see Table 4), diluted in 2% normal donkey serum in PBS with 0.3% Triton X-100 and the sections were exposed for 1 hour at room temperature. Negative controls consisted of both an unrelated isotype matched antibody, (see Table 3), or omission of the primary antibody. After the secondary antibody incubation, the sections were washed several times and the nuclei were subsequently stained with 0.5 ml 4′,6-diamidino-2-phenylindole (1 µl of DAPI stock solution, Sigma-Aldrich, to 5 ml of 0.1 M PBS) for 1 min. Slides were then washed several times with 0.1 M PBS followed by four washes in Tris buffered saline (pH 7.4) and finally glass coverslips were mounted in VECTASHIELD (Vector Laboratories). Sections were viewed using an Epi-fluorescence bright-field microscope (Olympus BX50F4, Olympus, Japan), where data was captured as 24 bit colour images at 3840×3072 pixel resolution using Nikon DXM1200 (Nikon,Tokyo, Japan) digital camera.

**Table 3:** Primary antibodies used for IHC detection in *cfh−/−* mouse retinae.

Primary antibody	Source	Catalogue number	Dilution
Mouse monoclonal antibody, 4G8, to amyloid beta (biotinylated)	Covance	SIG-39220 (SIG-39240)	1∶500 (1∶300)
Rat monoclonal antibody to mouse C3b/iC3b/C3cclone 2/11	Hycult Biotechnology	HM 1065	1∶50
Mouse IgG2a kappa [MOPC-173] isotype controlmonoclonal Ab	Abcam	ab18413	1∶100
Rabbit polyclonal to complement C3	Abcam	ab11887	1∶10
Anti-factor H goat polyclonal	Calbiochem, Merck Bioscience	341267	1∶50
Rabbit anti-rat polyclonal Ab to total C3	Hycult Biotechnology	HP8022	1∶50

**Table 4:** Secondary antibodies used for IHC detection in *cfh−/−* mouse retinae.

Secondaryantibody	Source	Catalogue number	Dilution
Alexa Fluor Donkeyanti Mouse 568	Invitrogen	A10037	1∶2000
Alexa Fluor Donkeyanti Rat 488	Invitrogen	A21208	1∶2000
Alexa Fluor Goatanti Mouse 488	Invitrogen	A21202	1∶2000
Alexa Fluor Goatanti Rat 594	Invitrogen	A11007	1∶2000
Alexa Fluor Goatanti Rabbit 488	Invitrogen	A21206	1∶2000
Alexa Fluor Donkeyanti Goat 568	Invitrogen	A10057	1∶2000
Alexa Fluor Donkeyanti Rabbit 594	Invitrogen	A21207	1∶500
Alexa FluorStreptavidin 488	Invitrogen	S11223	1∶500

### Grading System for IHC Analysis of the Retinae of *cfh−/−* mice

All IHC grading was performed in a masked manner by two experienced observers on four slides per eye, (both eyes per animal), with a minimum of four sections per slide (prophylactic regime), and five sections per slide, (therapeutic regime). For the baseline three month prophylactic group, this was only scored by one observer as at this stage little deposition of Aβ and activated complement C3 was found. The observer-based grading method was deemed more appropriate compared to morphological quantification due to the delicacy of sections and complexity of anatomy. An example of the grading system for the immunohistochemistry of retinal slides for Aβ, from the therapeutic regime is shown in Figure S1.

### Comparison of Systemic 6F6 Administration to an IgG2A Isotype Control in *cfh−/−* mice

In a separate study, *Cfh−/−* mice were treated at four months with a weekly dose of either 0.6 mg of 6F6 or an IgG2A isotype control, again via the intraperitoneal (IP) route for 12 weeks. Additionally, some animals in each group were dosed monthly with curcumin at 7.5 mg/kg i.v. to provide an alternative means of visualizing Aβ in hyperfluorescent foci [16]. At the end of the 12 weeks, animals from each group were similarly culled and eyes were processed for analysis by IHC. For this study, data for non-curcumin and curcumin-treated animals was pooled. The exact composition of all the groups in this study follows: For the 6F6 group n = 7 eyes, (n = 4 non curcumin treated, n = 3 curcumin treated), whereas for the IgG2A isotype control n = 6 eyes, (n = 2 non curcumin treated, n = 4 curcumin treated).

This study was performed by a different group of investigators to the remainder of the study and although the end-points were similar, some different methodology was used. Culling and ocular IHC was similarly performed. Retinal sections were blocked for 1 hr in 5% normal goat serum in 0.1 M phosphate buffer saline (PBS), pH 7.4 with 0.3% Triton X-100, before overnight incubation with primary antibodies. Although the same primary antibody was used for the detection of Aβ deposition as in the remainder of the study, (4G8, Table 3), this was biotin-labelled and detected with Alexa Fluor 488 streptavidin, and a different primary antibody was used to detect total C3, (Hycult biotech, cat# HP8022) a rabbit polyclonal anti-Rat C3 antibody which gave low background for IHC staining, (data from this study is described solely in the final part of the Results section and was the only occasion that the antibody combination described above was used in this work).

All IHC grading was similarly performed by two experienced observers in a masked fashion on two slides (six sections per slide) per animal/eye in each test group. A slight modification of the previous grading system was used to score IHC of the retinal slides and this is detailed in Methods S1.

### Statistical Analysis of Retinal Data Points after Treatment Regimes

The key endpoint data from the systemic administration of 6F6 and control administrations were subject to rigorous independent statistical analysis, (raw data from academic study investigators were evaluated by a GSK statistician, [PO]). For examination of the prophylactic regime, an analysis of variance was used to analyze data separately for IHC Aβ deposition level and IHC activated complement C3 levels. This model included a term for group and the eye was regarded as the experimental unit. For analysis of the IHC data: (i) where an analyst had scored an eye more than once, the mean score for that eye for that analyst was used, (ii) where more than one analyst had scored an eye, the mean of the analyst scores was used. For the therapeutic regime a similar analysis was done for all end-points but the additional terms for group, week and the group*week, interaction were used in the ANOVA model. Again a single eye was regarded as the experimental unit. Predicted group means were plotted, with 95% confidence interval. For the therapeutic regime, this was done for each week of endpoint analysis. Where analysis was performed on log10 scale, these data were back transformed to provide geometric means. Comparisons of group vs vehicle were made. For untransformed data, these are presented as differences from vehicle with 95% confidence interval and p values. For log transformed data, these are presented as ratios to vehicle with 95% confidence interval and p values. To assess the robustness of conclusions, a bootstrap test was performed. This simulates re-randomizations of eyes to new treatment groups 10,000 times, repeats the statistical analysis for each simulation, and compares the significance of the observed comparisons in this study against the range of possible results across simulations. The results are presented as bootstrap p value, as is the full statistical analysis of the key *in vivo* data which are also shown in Table form in the Supporting Information section. Data from the end-points of the prophylactic regime: Aβ IHC, (Table S1), activated complement C3, (Table S2); and from the therapeutic regime: Aβ IHC, (Table S3), activated complement C3, (Table S4) can be viewed in detail in the Supporting Information and the key significant findings are summarised in the Results section of the main text.

The data from ‘the comparison of systemic administration of 6F6 to an IgG2A isotype control in *cfh−/−* mice’, was similarly analysed as raw data by the same statistician, with some minor modifications in methodology. Where more than one analyst had scored a histological slide, the mean of the analyst scores was used. Where more than one histological slide had been scored for an eye, the mean score over the slides was used. The bootstrap analysis was not performed for the comparison of 6F6 v IgG2A isotype control.

### Immuno-assays and Statistics for PK and PD Analysis of Amyloid Beta in Mouse sera/plasma

Two immunoassays were designed to assess the pharmocodynamics of 6F6, (epitope 28–35), mediated transport of Aβ from the tissues to the periphery, [17], in *cfh−/−* mice. This was achieved by measuring free, (Aβ 1–40 and 1–42), and total, (free and antibody bound), Aβ 1–42, in the serum/plasma samples taken from treatment and control animals. This is summarized in Table 5. To measure free Aβ (non-drug bound) biotinylated 6F6 mAb, (4400 nM), was used as a capture reagent, and an Alexa Fluor 647-labelled Aβ-specific antibody, 4G8 (epitope 18–22), Covance (Cat# SIG39220), 1000 nM, was used as detection agent. To measure total Aβ 1–42, biotinylated Aβ-specific antibody 5G5 (specific for Aβ 1–42, C-terminal epitope 35–42), was used for capture and the alexa-labelled Aβ -specific antibody, 4G8 was used as detection. A 10 point standard curve was generated using human Aβ 1–42 peptide (Innogenetics NV, Cat No: 80315) ranging from 20000 ng/ml to 31.25 ng/ml in human Aβ depleted plasma, (GSK ‘in house’ reagent). Both of the described immunoassays were carried out on the Gyros AB Gyrolab xP workstation using the Bioaffy 1000 CDs, (Gyros Cat No: P0004253). All data generated for the Gyros immunoassays were analysed using the Gyrolab evaluator.

**Table 5:** Summary of peripheral amyloid beta detection assays.

Purpose	Capture antibody	Detection antibody	Comment
To detect free (not 6F6 bound) Aβ1–40 and Aβ 1–42	6F6 epitope (28–35)	4G8 epitope (18–22)	Ab epitopes can only confirm Aβ 18–35 detection
To detect total (free and 6F6 bound)Aβ 1–42	5G5 epitope (35–42)	4G8 epitope (18–22)	Ab epitopes can only confirm Aβ 18–42 detection

For statistical analysis of the data, free Aβ (1–40 and 1–42), and total Aβ 1–42 concentrations were log 10 transformed. Any values below assay LLOQ were imputed as LLOQ/2. Group means ±1 SD were calculated on the log scale and back transformed to proved geometric means ±1 SD range for bar plots. Analysis of variance of log 10 transformed data was used to estimate and compare geometric means with 95% confidence intervals. For the therapeutic study, comparisons were made on individual timepoints, (week 0, 4 and 12 post-administration). Comparisons are presented as ratios, with p values shown with/without multiple comparison adjustment using false discovery rate (FDR), [18].

## Results

### Characterisation of *cfh−/−* Mouse Model

The ocular phenotype of the the *cfh−/−* mouse at two years of age has been described [14], but the development of the ocular changes at earlier time-points, (three months and twelve months) has only be partially described, [19]. Further analysis of the *cfh−/−* mouse model was undertaken in this study to characterise the earlier development of an ocular phenotype that could be used to score an interventional study. Mice (n = 3), were also evaluated at 12 month old, (12MO) and an example of the retinal phenotypes displayed by *cfh−/−* mice is shown, in Figure 1. Large clusters of hyperfluorescent foci were present (Figure 1A). These autofluorescent points were easily distinguished from other autofluorescent structures as these patterns could also be seen in reflectance mode, (data not shown). The points were subsequently discovered to largely be present in the sub-retinal space, (Figure 1B–D). IHC labelled sections from the same age group revealed an abundance of partially digested fluorescent deposits, (yellow in the Figure), adjacent to the RPE, which contained activated forms of C3, (green-staining), labelled C3b+ (detected using clone 2/11, HM1065, Hycult Biotech., see Table 3), (Figure 1C), and are likely to be contained within macrophages, [20], [21]. The basal side of RPE showed extensive deposition of Aβ, (labeled green in Figure 1D), colocalising with the fluorescent debris (Figure 1D). Sudan Black had been used in the experiments shown in Figure 1 to dampen autofluorescent signals so that it was clear that positive IHC signals were genuine. A non-specific isotype control antibody was used to generate the negative control data, (Figure 1B). Similar hyperfluorescent spots have been seen in the retinae of C57Bl/6 mice though this is usually more pronounced at twenty four months and they have been identified as macrophages or microglia, [20]. Hyperfluorescent spots of macrophage origin have been seen in other transgenic mouse knockout models where the genotype accelerates the timing of their appearance with age, [21].

**Figure 2:**
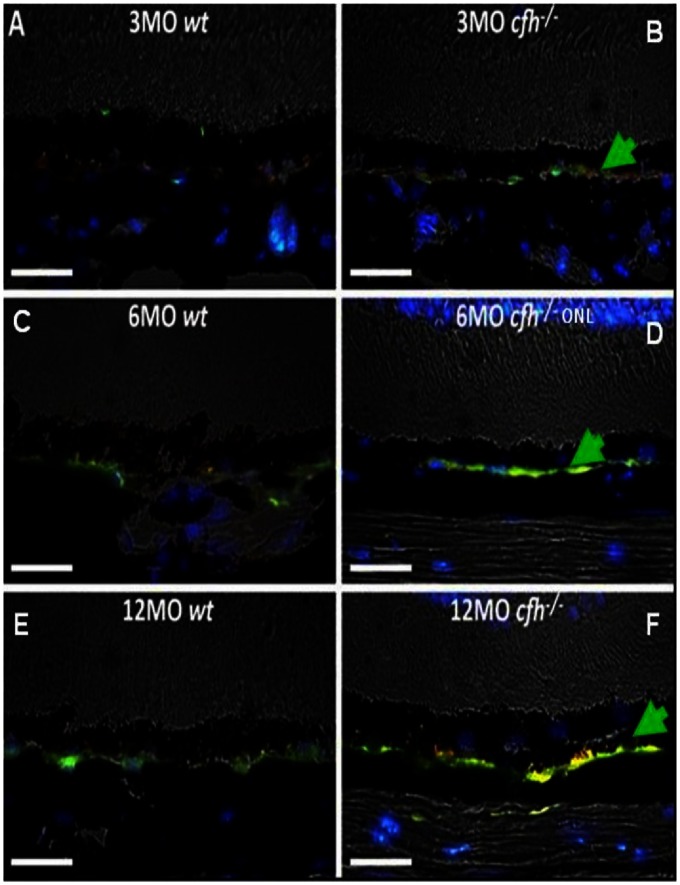
Longitudinal study of Aβ deposition along Bruch’s membrane in *wt* and *cfh−/−* mice. Aβ positive staining, (4G8+), is highlighted in green along the boundary of the retinal pigment epithelial, cells and Bruch’s membrane, (highlighted with a green arrow head), at the base of the retinae in the cfh−/− mouse eye. Aβ deposition increases with time in both *cfh–/−*, (B) 3 months, (D), 6 months and (F) 12 months, and the age­matched control (*wt*), retinae, (A) 3 months, (C), 6 months and (E) 12 months. Aβ deposition in *cfh−/–* mouse is elevated compared to wild type (*wt*) at similar ages. Note that there are some autofluorescent deposits, (yellow), interspersed below the RPE with the 4G8+ staining in the aged cfh−/− samples: (D) & (F). Blue label is DAPI, (4′,6-diamidino-2-phenylindole), a nuclear stain. Scalebar = 25 µm.

**Figure 3:**
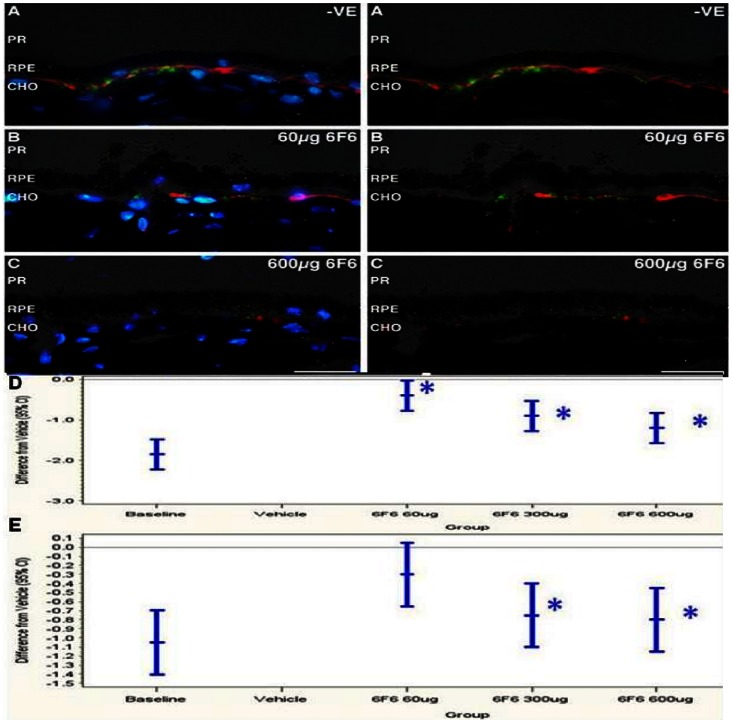
Immunohistochemical analysis of *cfh−/−* mice retinae after the prophylactic regime. Aβ (4G8+, red) and activated complement C3 (detected with clone 2/11+, #HM1065, Hycult Biotech., Table 3, green), cross reactivity to retinal sections at 6 months, (after 3 months dosing): (A) negative control (PBS treated), 6F6, at 60 µg dose (B) and 600 µg dose (C), scalebar = 25 µm, PR = phototreceptors/outer segments, RPE = retinal pigment epithelium, CHO = choroid, DAPI = 4',6-diamidino-2-phenylindole. Left hand panel (A), (B), (C) is stained with DAPI, right hand panel (A), (B), (C), is without DAPI staining. Aβ deposition (D) and activated complement C3 deposition (E) in the RPE/Bruch’s membrane and/or along the outer side of the choriocapillaris, (B), of *cfh−/−* mice are plotted as differences from vehicle control with 95% confidence intervals (CI), after prophylactic treatment in the test groups. Mice dosed with 6F6 show a dose dependent significant reduction, (asterisks, see text for levels of significance), in Aβ deposition at 60 µg, 300 µg and 600 µg and in activated C3 deposition at 300 µg and 600 µg compared to those treated with vehicle, (D) and (E) respectively.

**Figure 4:**
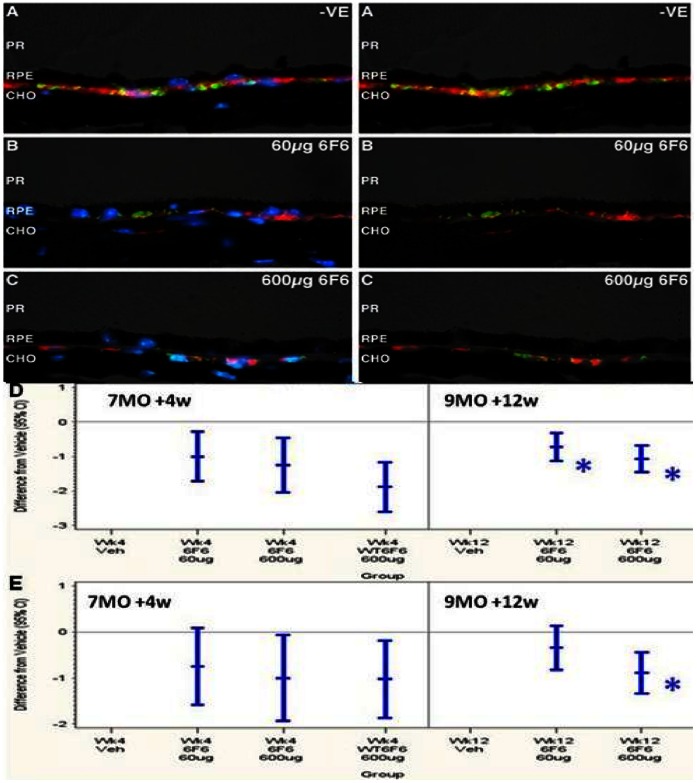
Immunohistochemical analysis of *cfh−/−* mice retinae of after the therapeutic regime. Aβ (4G8+, red) and activated complement C3 (detected with clone 2/11+, #HM1065, Hycult Biotech., Table 3, green), cross reactivity to retinal sections at 9 months, (after 3 months dosing): (A) negative control (PBS treated), 6F6, at 60 µg dose (B) and 600 µg dose (C). Scalebar = 25 µm), PR = phototreceptors/outer segments, RPE = retinal pigment epithelium, CHO = choroid, DAPI = 4',6-diamidino-2-phenylindole. Left hand panel (A), (B), (C) is stained with DAPI, right hand panel (A), (B), (C), is without DAPI staining. Aβ (D), and activated complement C3, (E), deposition in the RPE/Bruch’s membrane of *cfh−/−* mice, are plotted as differences from vehicle control with 95% confidence intervals (CI) after therapeutic treatment. Data in graphs (D) and (E) show data at an intermediate time point, after 7 months of age (7MO), 1 month (+4w) after treatment as well as at the end of the regime at 9 months of age (9MO), 3 months (+12w) after treatment. Data in graphs (D) and (E) also shows wild type (WT) mice treated with 6F6 (WT6F6) after 7 months of age (7MO), 1 month (+4w) after treatment. Mice dosed with 6F6 show a dose dependent significant reduction, (asterisks, see text for levels of significance), in Aβ deposition at 60 µg and 600 µg and in activated C3 deposition at 600 µg compared compared to those treated with vehicle, (D) at 9 months of age (9MO), 3 months (+12w) after treatment.

Using IHC for detection, Aβ+ staining appeared in retinae of 10 week-old cfh−/− mice (n = 3), on the basal side of the RPE, (data not shown). The appearance of Aβ coincided with the time at which complement C3, [19], and hyperfluorescent spots were first detected by IHC and scanning laser ophthalmoscope (SLO), respectively, (data not shown). Representative IHC from the retinae of wild type, C57Bl/6, (wt) and *cfh−/−* mice at 3 months, (3MO), 6 months, (6MO) and 12 months, (12MO) are shown in Figure 2. The data shows an increase in Aβ deposition, shown as 4G8+ Ab cross-reactivity, (Figure 2), with time in both *cfh−/−* and the age-matched control retinae. However, the rate of Aβ deposition in *cfh−/−* mouse is substantially increased compared to controls. The longitudinal analysis of Aβ deposition in the retina of the *cfh−/−* mouse matches that for the deposition of complement C3, [19]. IHC analysis has previously shown that complement factor H expression is predominantly found along Bruch’s membrane in the wild-type mouse, while in the *cfh−/−* mouse model C3 deposition accumulates with age on Bruch’s membrane [14]. Further analysis, had previously confirmed the deposition of activated C3 fragments, (C3b, iC3b and C3c), see also Figures 3 and 4, and presence along Bruch’s membrane in the aged *cfh−/−* mouse, [19] and for clarity this matches the timing of Aβ deposition, (Figure 2). From the time-course studies of the additional phenotyping of the *cfh−/−* mice performed, it was decided to nominally define the time-points for ‘prophylactic’ intervention in the model as three months, (just prior to the clear detection of Aβ and complement C3 deposition), and therapeutic intervention at six months, the point at which these end-points were clearly detectable, even in the absence of ocular functional differences in the *cfh−/−* mice at these times.

### Prophylactic Treatment Regime

#### IHC analysis and quantification of Aβ and activated complement C3 levels at the base of the retinae

At termination mice were culled, eyes were processed and IHC data generated from the prophylactic treatment regime was graded as described. Representative images are shown in Figure 3. Aβ deposition around the RPE/Bruch’s membrane, (red label), was present in the control group at 6 months (Figure 3A) but was clearly impacted by anti Aβ mAb treatment in the prophylactic regime (Figures 3B, 3C and 3D). The scoring of Aβ load and differences compared to the vehicle control group are shown in Figure 3D and in Table S1. Aβ was also, dependent upon fixation time, detected in some sections in outer segments and photoreceptors of *cfh−/−* mice at three and six months of age, without prophylactic treatment, (data not shown). Over-fixation can sometimes lead to 4G8 cross-reactivity to the photoreceptor layer, data not shown, but was not scored in this analysis and such staining is not seen in Fig 2. Cross-reactive staining of the photoreceptor layer with the 4G8 antibody is not a specific phenotype of *cfh−/−* mice but is also found in aged C57Bl/6 mice, but in both cases it is more pronounced at a later time of age, about 12 months, [20], (data not shown). The data demonstrated that: dosing with 6F6 significantly lowered the amount of Aβ deposited in the retinae of *cfh−/−* mice by scores of 1.2 (p<0.0001), 0.9 (p<0.0001) and 0.4 (p = 0.0378), at the 600 µg, 300 µg and 60 µg doses, respectively.

Activated complement C3 levels in the RPE/Bruch’s membrane were determined in a similar manner to that described for Aβ in IHC prepared retinal sections. Mean levels of activated complement C3 deposition, (green label, detected using clone 2/11, HM1065, Hycult Biotech., see Table 3), from 6F6 treated animals, (Figures 3B, 3C and 3E), compared to vehicle treated controls, (Figure 3A) are also shown in Figure 3. The scoring of activated complement C3 and differences compared to the vehicle control group are shown in Figure 3E and in Table S2. The data demonstrated that in addition to lowering retinal Aβ deposition: dosing with 6F6 significantly lowered the amount of activated complement C3 deposited in the retinae of *cfh−/−* mice by a score of 0.8 (p<0.0001), at a 600 µg dose, by a score of 0.75 (p<0.0001) at 300 µg dose, and by a score of 0.3 (p = 0.0938, non significant trend, at 60 µg dose).

### Therapeutic Treatment Regime

#### IHC analysis and quantification of Aβ and activated complement C3 levels at the base of the retinae

The IHC staining was graded according to the level of deposition of Aβ (4G8^+^) and activated complement C3, (C3b^+^) as described for the prophylactic regime. A representative image for both Aβ and activated complement C3 is shown in Figure 4. Aβ (red) and activated complement C3 deposition (green, detected using clone 2/11, HM1065, Hycult Biotech., see Table 3), around the RPE/Bruch’s membrane was strongly evident in the negative control group at nine months (Figure 4A) and was clearly impacted by 6F6 treatment in the therapeutic regime (Figures 4B, 4C and 4D). The scoring of Aβ load and differences compared to the vehicle control group are shown in Figure 4D and in Table S3.

Dosing with 6F6 at 600 µg significantly lowered Aβ deposited in the retinae of *cfh−/−* mice by a score of 1.25 (p = 0.0028) at week 4, and by 1.06 points at week 12 (p<0.0001). Dosing with 6F6 at 60 µg showed a significant lowering of the amount of Aβ deposited in the retinae of *cfh−/−* mice by a score of 1 (p = 0.0069) at week 4 and significantly lowered score by 0.72 (p = 0.0008) at week 12. Mean levels of Aβ deposition in C57Bl/6 mice after 4wks treatment with 6F6, (600 µg) showed a reduction over those in *cfh−/−* mice after the same treatment, (Figure 4D and Table S3), which fits the lower rate of Aβ deposition in wild type mice, Figure 2, [20].

Activated complement C3 levels, (detected using clone 2/11, HM1065, Hycult Biotech., see Table 3), in the RPE/Bruch’s membrane were determined in a similar manner to that described above for Aβ. Activated complement C3 deposition differences compared to vehicle treated controls are shown in Figure 4E and in Table S4. The data demonstrated that: dosing with 6F6 at 600 µg significantly lowered the amount of activated complement C3 deposited in the retinae of *cfh−/−* mice by a score of 1.00 points at week 4 (p = 0.0379) and by 0.89 (p = 0.0002) at week 12; dosing with 6F6 at 60 µg showed a non significant trend towards lowering the amount of activated complement C3 deposited in the retinae of *cfh−/−* mice by a score of 0.75 (p = 0.0798) at week 4 and by a score of 0.34 (p = 0.1594) at week 12. Mean levels of activated complement C3 deposition in C57Bl/6 mice after 4wks treatment with 6F6, (600µg) showed a similar trend for reduction to that seen in *cfh−/−* mice after the same treatment, (Figure 4E and Table S4).

### Immuno-assays for PK and PD Analysis of Aβ in Mouse Sera

#### Quantification of unbound (free) Aβ

Data showing these analyses in summary are displayed in Figure S2 & Table S5 (Prophylactic regime) and Figures S3 & S4 & Table S6 (Therapeutic regime).

*Cfh−/−* mice treated prophylactically with 6F6 showed a large reduction in free, (non-antibody bound) Aβ 1–40/1–42 compared to controls, using the 6F6-4G8 Gyros assay. This is statistically significant for all 6F6 dose groups but the 300 µg dosed group showed more variability between individual serum samples and the dose response is unclear.

*Cfh−/−* mice treated therapeutically with 6F6 at 600 µg dose showed a small reduction in free, (non-antibody bound), Aβ 1–40/1–42 levels in plasma, after 4 weeks treatment which was almost statistically significant when compared to PBS controls, (see Figures S3 & S4 & Table S6). Free Aβ concentrations in the 6F6 treated animals were not substantially reduced compared to the PBS controls at the 12 week time point.

#### Quantification of total Aβ 1–42

**Figure 5:**
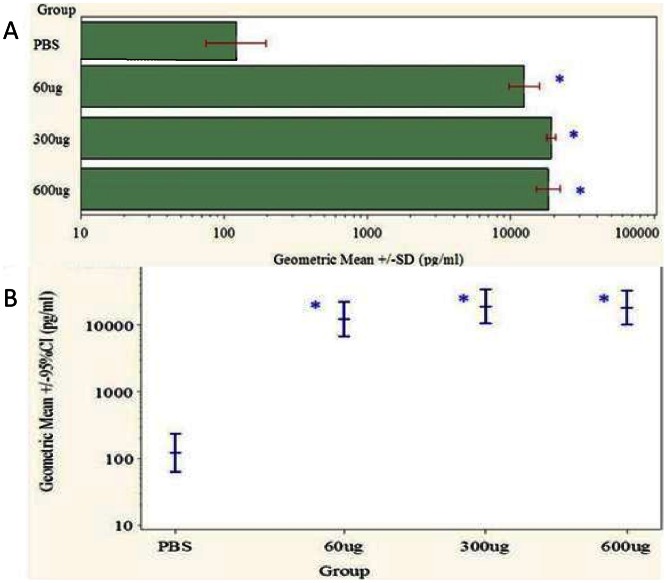
Total Aβ 1–42 levels in *cfh−/−* mouse sera after prophylactic administration regime. Concentration of total Aβ 1–42 in serum samples treated systemically are shown as geometric means with standard deviation, (A) and with 95% Confidence Intervals from statistical analysis (B). Data is shown at the end of the prophylactic regime, (6 months, after 3 months treatment), for n = 5 mice per treatment group, 6F6 dosed unless stated. Note the substantial increases in serum total Aβ 1–42 after 6F6 dosing, (see text for details)**.** Statistical significance, p<0.0001, (FDR adjusted), was reached for all doses of 6F6 over PBS controls, see Table S7.

The data in Figure 5 and Figure S5 and Table S7 show the geometric mean total serum Aβ 1–42 concentrations determined for treated *cfh−/−* mouse groups at the end of the prophylactic study. In 6F6 treated animals, at all doses, there was a dramatic, statistically significant, (p<0.0001, FDR adjusted), increase in total, (free and bound), Aβ 1–42 when compared to PBS-treated controls, which appears to reach saturation at 300 µg dose and above, (Fig 5A & 5B).

**Figure 6:**
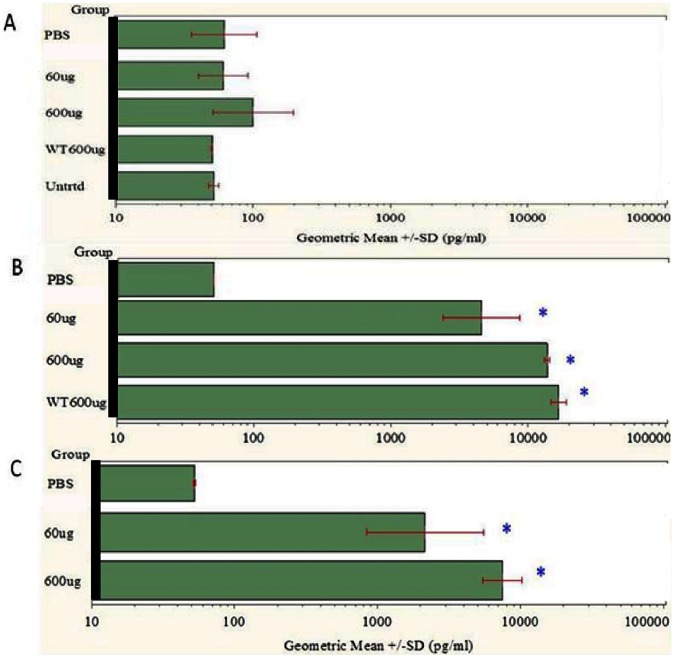
Total Aβ 1–42 levels in *cfh−/−* mouse plasma after therapeutic administration regime. Concentration of total Aβ 1–42 in plasma samples treated systemically are shown as geometric means with standard deviation at baseline (A), (n = 12/group) and after 4 weeks (B), (n = 4/group), and 12 weeks (C) of the therapeutic regime where final numbers were: PBS, vehicle n = 6; 6F6, 60 µg and 600 µg, n = 5. Labels are 6F6 dosed unless stated, Untrtd = untreated *cfh−/−* mice, WT600 µg = C57Bl/6 mice dosed with 6F6. Note the substantial increases in total plasma Aβ 1–42 after 6F6 dosing in *cfh−/−* and C57Bl/6 mice at both the 4 (B) and 12 (C) week time-points, (see text for details)**.** Statistical significance, p<0.0001, (FDR adjusted), was reached for all doses of 6F6 over PBS controls, see Table S8.

The mean plasma total Aβ 1–42 concentrations for all 6F6 dosed animals, (both *cfh−/−* and C57Bl6), in the therapeutic study, (see Figure 6 and Table S8), at the two time-points of 4 (Fig. 6B), and 12 weeks (Fig. 6C) show a dramatic statistically significant, (p<0.0001, FDR adjusted), increase in total, (free and bound), Aβ 1–42 compared to PBS-treated controls. This increase was dose-dependent although not dose-proportional. The mean increase over baseline values observed for both the 60 µg and 600 µg 6F6 *cfh−/−* treatment groups at 12 weeks, (Figs. 6A & 6C), was lower than the values seen at the 4 week time-point, (Figs. 6A & 6B). This may indicate a long term depletion of Aβ 1–42, from the system at these high, weekly, systemic doses. *Cfh−/−* mice have normal murine amyloid precursor protein, (APP) expression and therefore have normal Aβ 1–42, levels, unlike the situation in transgenic mice which over-express human APP in the brain, (so-called AD Tg mouse models), [22]. Consequently, the increases seen in the wild type C57Bl/6 mice treated with 6F6 at 600 µg are comparable to those seen in the *cfh−/−* mice after 4 weeks, (Fig. 6B).

### Comparison of Systemic Administration of 6F6 to an IgG2A Isotype Control in *cfh−/−* mice

To address concerns that vehicle alone may not be an adequate negative control for the previous experiments, a further study was performed on 4 month-old *cfh−/−* mice. The new study compared the impact on the previously scored ocular measures of animals treated with 600 µg 6F6 run ‘head to head’ with those treated with 600 µg of an IgG2A isotype control on a similar weekly systemic administration regime.

**Figure 7:**
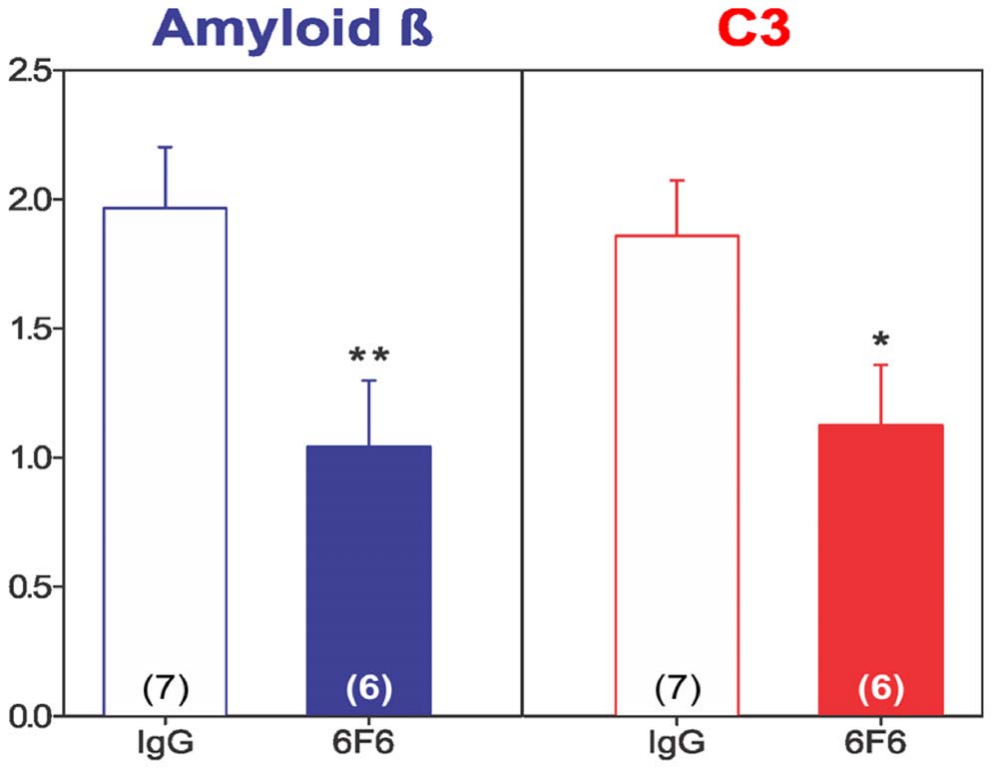
Immunohistochemical analysis of *cfh−/−* mice retinae after treatment with 6F6 v IgG2A isotype control Abs. Log scored means +/− SE for Amyloid β (4G8+, blue) and complement C3 (C3+, red, detected with rabbit anti-rat polyclonal Ab to total C3, Hycult, HP80222, Table 3), deposition in retinal sections after 3 months of dosing with: (i) 6F6, [solid bars]; (ii) IgG, IgG2A isotype specific negative control Ab, [open bars], at 600 µg weekly doses. Group sizes, n = numbers of eyes treated, are marked at the base of the bars in parentheses. Differences of **p = 0.0232, for Amyloid β deposition and of *p = 0.0414 for C3 deposition were noted comparing 6F6 treated with IgG2A control. See SI Figure S6.

From the analysis of both Aβ and complement C3 deposition around the RPE/Bruch’s membrane, the IHC score ranges for the two subgroups +/− curcumin overlapped, (see Figure S6) within each treatment group so it was considered a reasonable approach to pool the sub-groups for statistical analysis. The comparative data for both Aβ and complement C3 deposition is shown in Figure 7, where it can be seen that treatment with 6F6 reduces deposition of both Aβ (p = 0.0232) and complement C3, (p = 0.0414), with statistical significance, when compared to treatment with an IgG2A isotype control. Note that in this study, total C3, (ie. both activated and uncleaved C3), was being detected using a rabbit polyclonal anti-Rat C3 antibody, (Hycult biotech, cat# HP8022, Table 3), and yet statistical significance for co-clearance of Aβ and complement C3 was still demonstrated by IHC.

## Discussion

Prophylactic treatment of *cfh−/−* mice with 6F6 led to a dose dependent, (3–30 mg/kg), reduction in the retinal deposition of Aβ and activated complement C3. Therapeutic treatment of *cfh−/−* mice with 6F6 at a 30mg/kg dose led to a significant reduction in the retinal deposition of Aβ and activated complement C3 after both 4 and 12 weeks. Data obtained on retinal levels of Aβ and activated complement C3 were very carefully scored, mindful that RPE/Bruch membrane derived autofluorescence did not impact the interpretation of the results.

Levels of total Aβ 1–42 peptide in the periphery of 6F6 treated animals were dramatically increased compared to controls, after both the prophylactic and therapeutic regimes, and this was dose dependent. In animals treated therapeutically with 6F6 the increase in mean peripheral Aβ 1–42 levels was greater after 4 weeks than 12 weeks. This may be a result of depletion of systemic Aβ 1–42 peptide by the repeat dose antibody treatment. Taken together, these data are in line with the concept that soluble Aβ available in the blood is effectively bound by 6F6 and that 6F6 may have Aβ lowering effects on other compartments in the animal, such as the eye, through the hypothesized peripheral sink mechanism, increasing the total Aβ in the blood stream [17], [23]. Similar results have been obtained from plasma analysis from systemically treating transgenic hAPP mice with 6F6 at comparable doses, (unpublished data). A repeat experiment, initiated three years after the original study by a different group of investigators was performed in the *cfh−/−* mice to compare the effect of 6F6 treatment in parallel to treatment with a similar dose of an IgG2A isotype control. The data generated from this study verified the reduction in deposition of both Aβ and complement C3 after systemic treatment with 6F6 but not with an IgG2A isotype control antibody and this was statistically significant in both cases.

Recently published data suggest that Aβ deposition occurs with age even in the retina of wild type C57Bl/6 mice and that the phenotype of the *cfh−/−* mouse is super-imposed on an accelerated version of what might occur at a later time-point in wild type mice, [20]. In wild type mice similar hyperfluorescent spots were described to those seen in *cfh−/−* mice and it was shown that these correspond to microglial cells that contain Aβ, [20]. Further aspects of the accelerated *cfh−/−* ocular phenotype may be related to exposure of the mouse model to external pathogens, [24].

The eyes of aged, targeted-replacement apoE mice expressing human Apo E4, (HuApoE4 KI), when placed on a high fat diet developed changes which mimic AMD pathology with diffuse sub-RPE deposits, Bruch’s membrane thickening, RPE atrophy, both hypopigmentation and hyperpigmentation of the RPE [3]. In some cases mice develop marked CNV and there is loss of retinal function as measured by electroretinogram (ERG), [3]. The HuApoE4 KI mouse model also demonstrates the presence of murine Aβ both associated with the CNV and with the outer retinal deposits and the presence of elevated levels of murine VEGF, [3]. The HuApoE4 KI mouse has been used to test the hypothesis that the intraperitoneal injection of the Aβ mAb, 2H6, can be used to reduce the load of outer retinal deposits in a similar way to the reduction of Aβ containing plaques in the brains of AD models. Preliminary evidence suggested that Aβ basal deposits were reduced in these mice upon systemic administration of anti-Aβ mAbs and that ERGs could be partially restored, [23, 25, 26, 27 & 28]. It is notable that in the HuApoE4 model 2H6 which also only binds Aβ 1–40 and not Aβ 1–42 was only partially protective [27], [28]. A later study published by the same group, demonstrated the ability of systemically administered anti-Aβ mAbs to additionally lead to a reduction in levels of activated complement C3 from the retina of HuApoE4 mice, similar to here in *cfh−/−* mice [28]. The most effective anti-Aβ mAb in the second HuApoE4 study was RN6G which has high affinity for both Aβ 1–40 and Aβ 1–42, similar to 6F6. Note that 6F6 shows high affinity, single digit nM, binding for both Aβ 1–40 and Aβ 1–42 and both a fast on rate and a slow off rate for both peptides by SPR, (data not shown). The accumulation of Aβ in the periphery of mice with essentially normal levels of rodent Aβ, (rather than mice over-expressing human Aβ), is demonstrated here in *cfh−/−* mice, after systemic administration of anti-Aβ mAbs and was also recently published for the HuApoE4 model, [28]. The *cfh−/−* mouse may represent a rapid model to test the impact of therapies on clearance of activated complement C3 and Aβ deposition, however, *cfh−/−* mice only have minor visual defects and these are more apparent at two years of age, [14]. Attempts were made to analyse a small sub group of negative control animals and high dose 6F6 treated animals, from the therapeutic regime, for differences in retinal function by ERG, (data not shown). There was no clear difference between control and treated groups for the scotopic, a- & b-waves, however data for the phototopic, a- & b- waves appeared to show a a trend in favour of treated animals in this very small sample group, (data not shown). It was deemed impractical to dose *cfh−/−* mice weekly from three to twenty-four months of age with high doses of 6F6 to fully impact retinal function readouts in this model, [14].

The link between the aetiology of AMD and AD has been thoroughly reviewed recently [29]. While reducing amyloid burden remains a key issue in both ageing and disease, it is important to note that some forms of amyloid are critical for normal biological function including synaptic plasticity and memory [30], [31]. Initial studies to characterize Aβ in drusen, appeared to highlight some differences from the Aβ found in AD plaques. The conclusions from these studies were that drusen exhibit some of the characteristics of AD plaque Aβ and contains several Aβ associated proteins but not Aβ fibrils. However, one study was unable to detect Aβ peptide, nor amyloid precursor protein, (APP) in drusen, [32]. This was in contrast to an early study that had reported the reaction of drusen with monoclonal antibodies directed against Aβ peptide, [33]. Further very detailed characterisation of drusen found Aβ to associate with a sub-structural vesicules co-localizing with activated complement components C3, [34], [35]. Aβ could be an important component of the local inflammatory events that lead to RPE atrophy, drusen biogenesis and the pathogenesis of AMD. Aβ-containing drusen were also studied in 152 donor human eyes. Donors with AMD possessed some drusen with the Aβ assemblies. Vesicles were sometimes found in the process of budding or fusing. Aβ immunoreactivity was also found in the cytoplasm of RPE cells [35].The presence of Aβ was confirmed in drusen and the expression of APP, its progenitor, was highlighted in RPE cells using a number of antibody reagents with documented binding activity in AD plaques, [10].

Structures similar to the drusen associated ‘amyloid vesicles’ have been described in brains of transgenic mice expressing the human APP protein [35]. A study to look at Aβ in the drusen of AMD compared to normal retinas found immunoreactivity in 4/9 AMD eyes and 0/9 normal eyes [36]). Further studies detected oligomeric Aβ in drusen which did not co-localize with ‘amyloid vesicles’ [37]. Oligomeric Aβ reactivity was seen in all drusen but not in eyes without drusen. A recent study, [38], has shown a variety of Aβ stuctures in drusen vesicles including: non-fibrillar oligomers, protofibrils and mature Aβ of which non-fibrillar oligomers appear to be the most abundant, [34]. Aβ in drusen could contribute to AMD by assembling into macromolecular aggregates containing cytotoxic Aβ peptide forms resulting in direct killing of RPE and/or retinal ganglion cells [34]. Aβ directly interacts with VEGF and this may play a role in AD and AMD pathogenesis [39]. Activation of the alternative complement pathway triggers VEGF expression, but Aβ can also induce VEGF expression in human RPE cells *in vitro* [5].

A study of the prevalence of AMD amongst AD patients in the USA found approximately double the number of expected cases, [40]. Conversely a prospective population-based study identified an increased risk of developing AD in individuals with advanced AMD, [41]. However, a similar analysis of AMD levels performed on participants in a cardiovascular health study concluded that there was no significant association between AD and early AMD, [42]. A recent study, [43], suggested an interaction between the common AMD-associated CFH polymorphism Y402H, and the APOE E4 allele together pre-disposing patients for co-morbidity in AD and AMD. However, the Y402H polymorphism in the CFH gene was not significantly associated with AD in two further studies, [44 & 45].

Recently published data has shed light on the interaction of complement proteins and Aβ in the generation of AMD [46 & 47]. Aβ has been shown to bind to complement factor I, (CFI), the co-factor that with factor H is responsible for the breakdown of complement protein C3 from the C3b form to its inactive form, iC3b, [46]. These results support a hypothesis where Aβ activates the complement system within drusen by blocking the function of CFI, leading to a low-grade, chronic inflammation in sub-retinal tissues linking four of the factors associated with the development of AMD: inflammation, complement activation, Aβ deposition and drusen, [46]. Both Aβ 1–40, [48], and Aβ 1–42 [49], peptides have been shown to induce inflammatory responses when localizing to the rat retina after intravitreal injection, in the case Aβ 1–40 it has been shown to activate the NLRP-3 inflammasome, [48]. Aβ has been shown both to increase the expression of monocyte chemo-attractant protein-1, (MCP-1), in RPE cells and to elevate the production of IL-1β and TNFα from macrophage and microglial cells *in vitro*, [47]. The combined effect of co-culturing macrophages and RPE cells with Aβ is therefore to up-regulate complement factor B, CFB, expression from RPE cells, [47]. Aβ could therefore have a combined mechanism of CFB activation and CFI inhibition to activate the alternative complement pathway and accelerate the progression of AMD. Aβ co-localisation, interaction with and binding to iC3b and other complement C3 components has been well-documented, [12], [29]. One possible explanation for the co-clearance, after 6F6 administration, in the *cfh−/−* mouse retina of Aβ and activated complement C3, is that complement is removed to the periphery, bound to Aβ. Note that Aβ and activated complement C3 deposition in the retina of *cfh−/−* mice have a very similar time course of appearance.

In summary, this work provides further evidence that Aβ may be a key factor in AMD pathology and disease. The exact mechanisms which cause the production of Aβ from RPE and the exact mechanism or mechanisms by which Aβ acts to influence AMD are not understood. However, current evidence implies that clearing of Aβ by agents that bind and potentially neutralise or just remove Aβ may provide a possible route to clearing drusen in AMD, reducing complement activation, reducing RPE atrophy and potentially reducing the induction of VEGF expression in RPE and its localization at high levels around drusen. Such therapy could therefore provide means of preventing, delaying, attenuating or reversing the loss of vision due to AMD and its progression to geographic atrophy and/or exudative AMD. This may result in decreased levels of Aβ and activated complement C3 containing drusen and/or local Aβ in the surrounding environment of the RPE and thereby interfere in both the early and later stages of AMD and treat the underlying cellular decline that causes the loss of vision.

## Supporting Information

Figure S1 Representative grading of the retinae of *cfh−/−* mice in the therapeutic regime scored for the level of Aβ deposition by immunohistochemistry (IHC). Aβ staining is in red (4G8+) and is indicated by white arrows. Blue label is DAPI, (4′,6-diamidino-2-phenylindole) a nuclear stain. Scalebars = 25 µm. ONL = Outer nuclear layer, PR = photoreceptors, RPE/BR = retinal pigment epithelium/Bruch’s membrane, CHO = choroid. Grading protocol for IHC: Grade 0, No deposition along the Bruch’s Membrane, (A), Grade 1, Fragmented deposition or <10% deposition along Bruch’s membrane, (B), Grade 2, Segmental deposition along 10–50% of Bruch’s membrane, (C), Grade 3, Close to continuous deposition, or 50–75%, along the length of Bruch’s membrane, (D), Grade 4, A continuous expression, or >7 5% of Bruch’s membrane, (data not shown).
(TIF)

Figure S2 Free Aβ (1–40 & 1–42) levels in *cfh−/−* mouse sera after prophylactic administration regime. Concentration of free Aβ (1–40 &1–42) in serum samples are shown as geometric means with standard deviation, (A) and with 95% Confidence Intervals (B) Data are shown at the end of the prophylactic regime, (6 months, after 3 months treatment), for n = 5 mice per treatment group, 6F6 dosed unless stated. Note the substantial decreases in serum free Aβ (1–40 & 1–42) after 6F6 dosing, (see text for details). Statistically significant, (FDR adjusted), differences were reached for 6F6 dosed animals over the PBS-dosed controls of p = 0.0003, (60 µg), p = 0.0175, (300 µg) p = <0.0001, (600 µg), see Table S5.
(TIF)

Figure S3 Free Aβ (1–40 & 1–42) levels in *cfh−/−* mouse plasma after therapeutic administration regime. Concentration of free Aβ (1–40 &1–42) in plasma samples are shown as geometric means with standard deviation at baseline (A), (n = 12/group) and after 4 weeks (B), (n = 4/group), and 12 weeks (C) of the therapeutic regime where final numbers were: PBS, vehicle n = 6; 6F6, 60 µg and 600 µg, n = 5. Labels are 6F6 dosed unless stated, Untrtd = untreated *cfh−/−* mice, WT600 µg = C57Bl/6 mice dosed with 6F6. Note a drop in plasma free Aβ (1–40 & 1–42) levels at a 600 µg dose of 6F6 at the 4 week time-point, which is close to statistical significance, over PBS controls p = 0.1416, (FDR adjusted), p = 0.0354 (non-adjusted), see Table S6.
(TIF)

Figure S4 Geometric mean free Aβ (1–40 & 1–42) levels with 95% Confidence Intervals in *cfh−/−* mouse plasma after therapeutic administration regime.
(TIF)

Figure S5 Geometric mean total Aβ 1–42 levels with 95% Confidence Intervals in *cfh−/−* mouse plasma after therapeutic administration regime.
(TIF)

Figure S6 Comparison of relative spread of immunohistochemical scoring of the retinae of *cfh−/−* mice after treatment with 6F6 v IgG2A isotype control Ab. Data for geometric mean values of IHC scores for Amyloid β (4G8 = analyte): (A) scatter, (B) box & tail, and complement C3, detected with rabbit anti-rat polyclonal Ab to total C3, Hycult, HP80222, Table 3, (C3 = analyte): (C) scatter, (D) box & tail; were compared across the groups treated with either 6F6 or IgG2A and further sub-divided into sub-groups scored by standard autofluorescence, (AF, not further treated) and those additionally dosed with Curcumin, (CU). For the scatter plots: (A) & (C), a small constant greater than one was added to the data to highlight any overlapping points that might mask the true analysis of variability. For the 6F6 treated n = 7 eyes, (n = 4 AF, non curcumin treated, n = 3 curcumin treated) and for the IgG2A isotype control n = 6 eyes, (n = 2 AF, non curcumin treated, n = 4 curcumin treated). The IHC score ranges for the two subgroups +/− curcumin overlapped within each treatment group so it was considered a reasonable approach to pool the sub-groups for statistical analysis.
(TIF)

Table S1 Mean levels (A) and ratios to vehicle control group (B) of Aβ deposition in the RPE/Bruch’s membrane after prophylactic treatment.
(TIF)

Table S2 Mean levels of activated complement C3 deposition (A) and ratios to vehicle controls (B) in the RPE/Bruch’s membrane after prophylactic treatment.
(TIF)

Table S3 Mean levels (A) and ratios to vehicle control group (B) of Aβ deposition in the RPE/Bruch’s membrane after therapeutic treatment.
(TIF)

Table S4 Mean levels (A) and ratios to vehicle control group (B) of activated complement C3 deposition in the RPE/ Bruch’s membrane after therapeutic treatment.
(TIF)

Table S5 Geometric means of free Aβ 1–40/1–42 levels for *cfh−/−* mice at the end of the prophylactic regime in sera (A) and statistical analysis (B).
(TIF)

Table S6 Geometric means of free Aβ 1–40/1–42 levels for *cfh−/−* mice at the end of the therapeutic regime in plasma (A) and statistical analysis (B).
(TIF)

Table S7 Geometric means of total Aβ 1–42 levels for *cfh−/−* mice at the end of the prophylactic regime in sera (A) and statistical analysis (B).
(TIF)

Table S8 Geometric means of total Aβ1–42 levels for *cfh−/−* mice at the end of the therapeutic regime in plasma (A) and statistical analysis (B).
(TIF)

Methods S1 Comparison of systemic administration of 6F6 to an IgG2A isotype control in *cfh−/−* mice using Curcumin as an imaging marker.
(DOCX)
