# Supplementary figures and images for: Systemic Administration of Abeta mAb Reduces Retinal Deposition of Abeta and Activated Complement C3 in Age-Related Macular Degeneration Mouse Model
Source: PLoS One. 2013 Jun 14;8(6):e65518. doi: 10.1371/journal.pone.0065518 (PMC3682980; doi:10.1371/journal.pone.0065518)

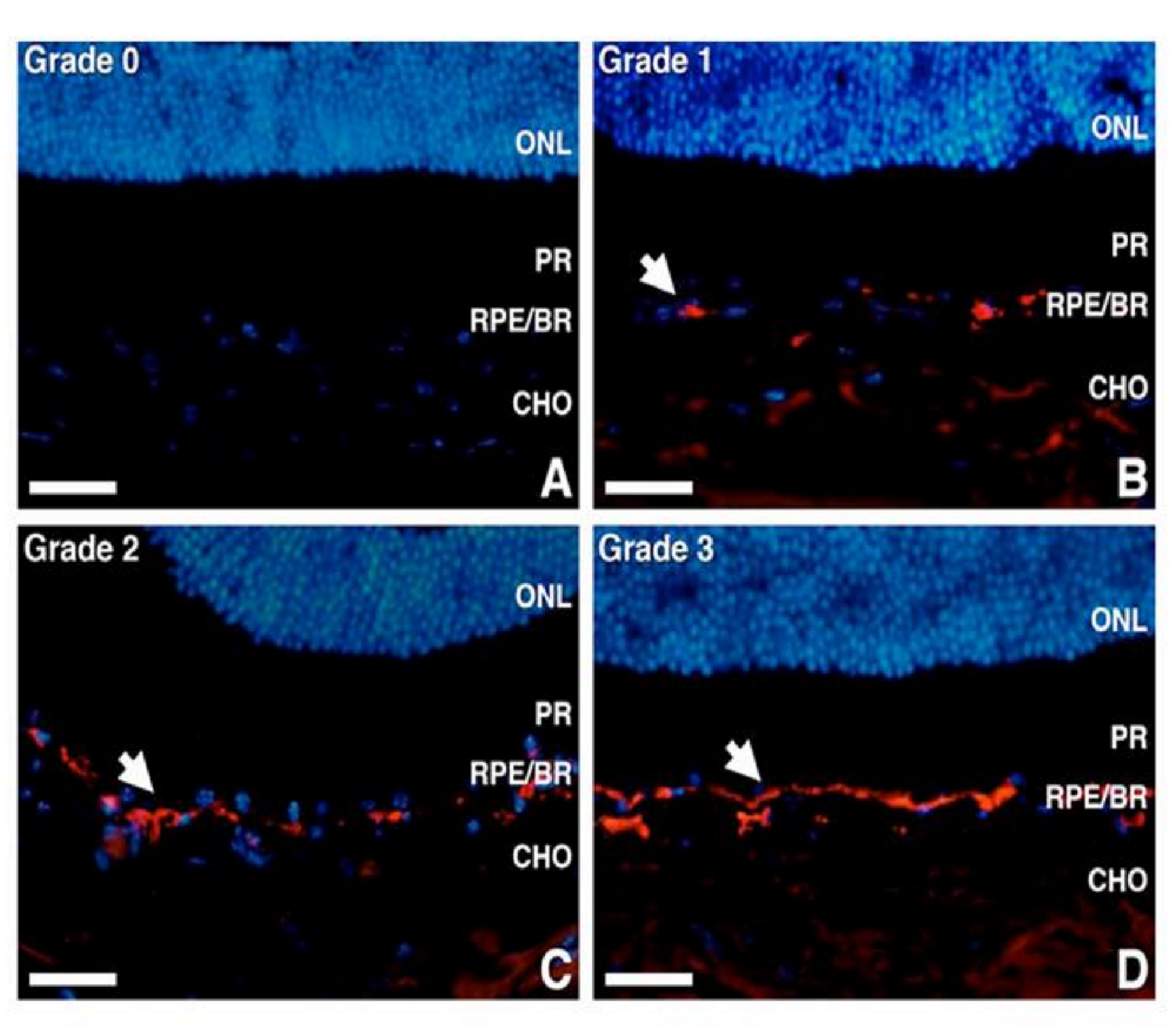

Supplement: Figure S1 — Representative grading of the retinae of cfh−/− mice in the therapeutic regime scored for the level of Aβ deposition by immunohistochemistry (IHC). Aβ staining is in red (4G8+) and is indicated by white arrows. Blue label is DAPI, (4′,6-diamidino-2-phenylindole) a nuclear stain. Scalebars = 25 µm. ONL = Outer nuclear layer, PR = photoreceptors, RPE/BR = retinal pigment epithelium/Bruch’s membrane, CHO = choroid. Grading protocol for IHC: Grade 0, No deposition along the Bruch’s Membrane, (A), Grade 1, Fragmented deposition or <10% deposition along Bruch’s membrane, (B), Grade 2, Segmental deposition along 10–50% of Bruch’s membrane, (C), Grade 3, Close to continuous deposition, or 50–75%, along the length of Bruch’s membrane, (D), Grade 4, A continuous expression, or >7 5% of Bruch’s membrane, (data not shown). (TIF) [file pone.0065518.s001.tif]

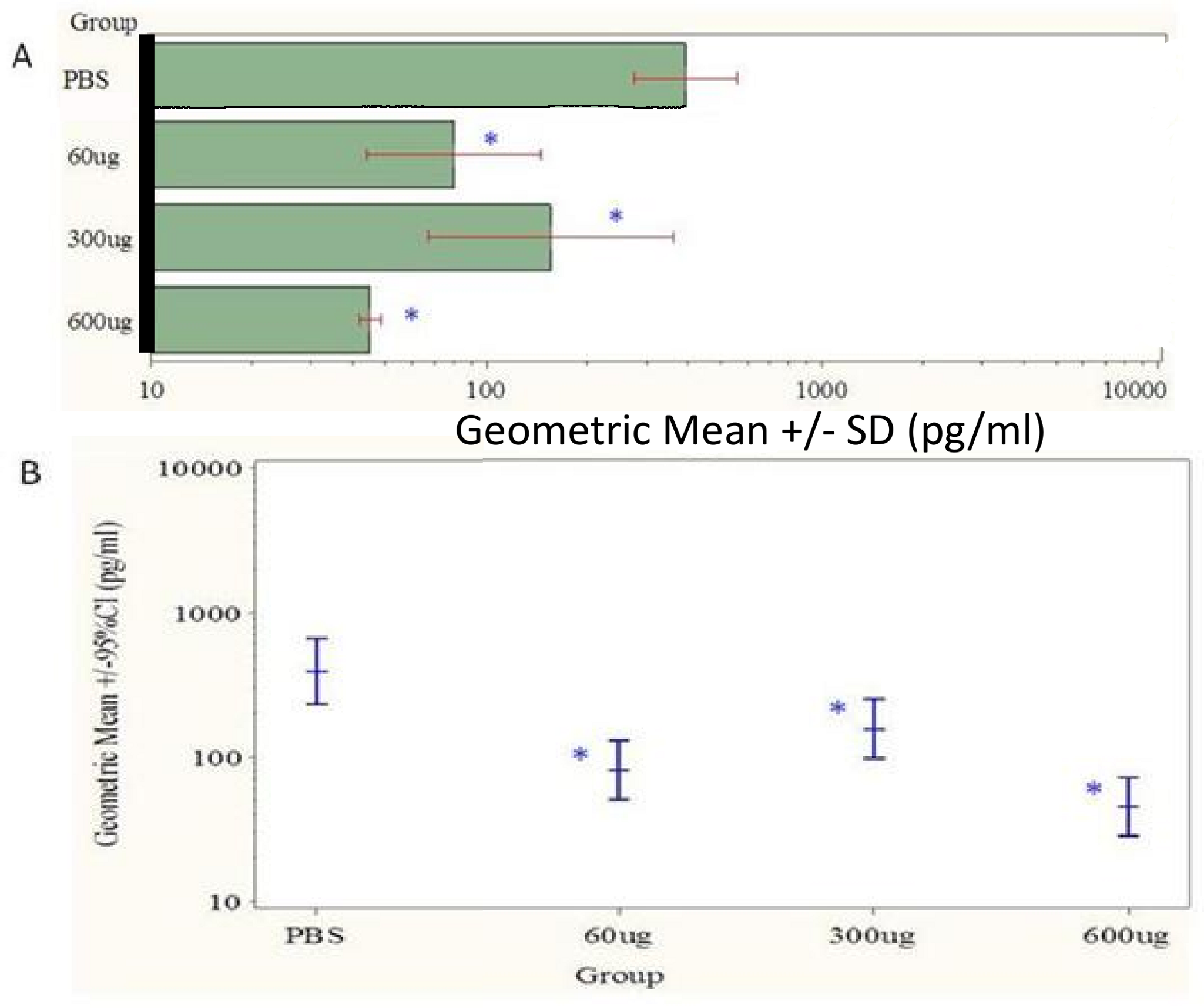

Supplement: Figure S2 — Free Aβ (1–40 & 1–42) levels in cfh−/− mouse sera after prophylactic administration regime. Concentration of free Aβ (1–40 &1–42) in serum samples are shown as geometric means with standard deviation, (A) and with 95% Confidence Intervals (B) Data are shown at the end of the prophylactic regime, (6 months, after 3 months treatment), for n = 5 mice per treatment group, 6F6 dosed unless stated. Note the substantial decreases in serum free Aβ (1–40 & 1–42) after 6F6 dosing, (see text for details). Statistically significant, (FDR adjusted), differences were reached for 6F6 dosed animals over the PBS-dosed controls of p = 0.0003, (60 µg), p = 0.0175, (300 µg) p = <0.0001, (600 µg), see Table S5. (TIF) [file pone.0065518.s002.tif]

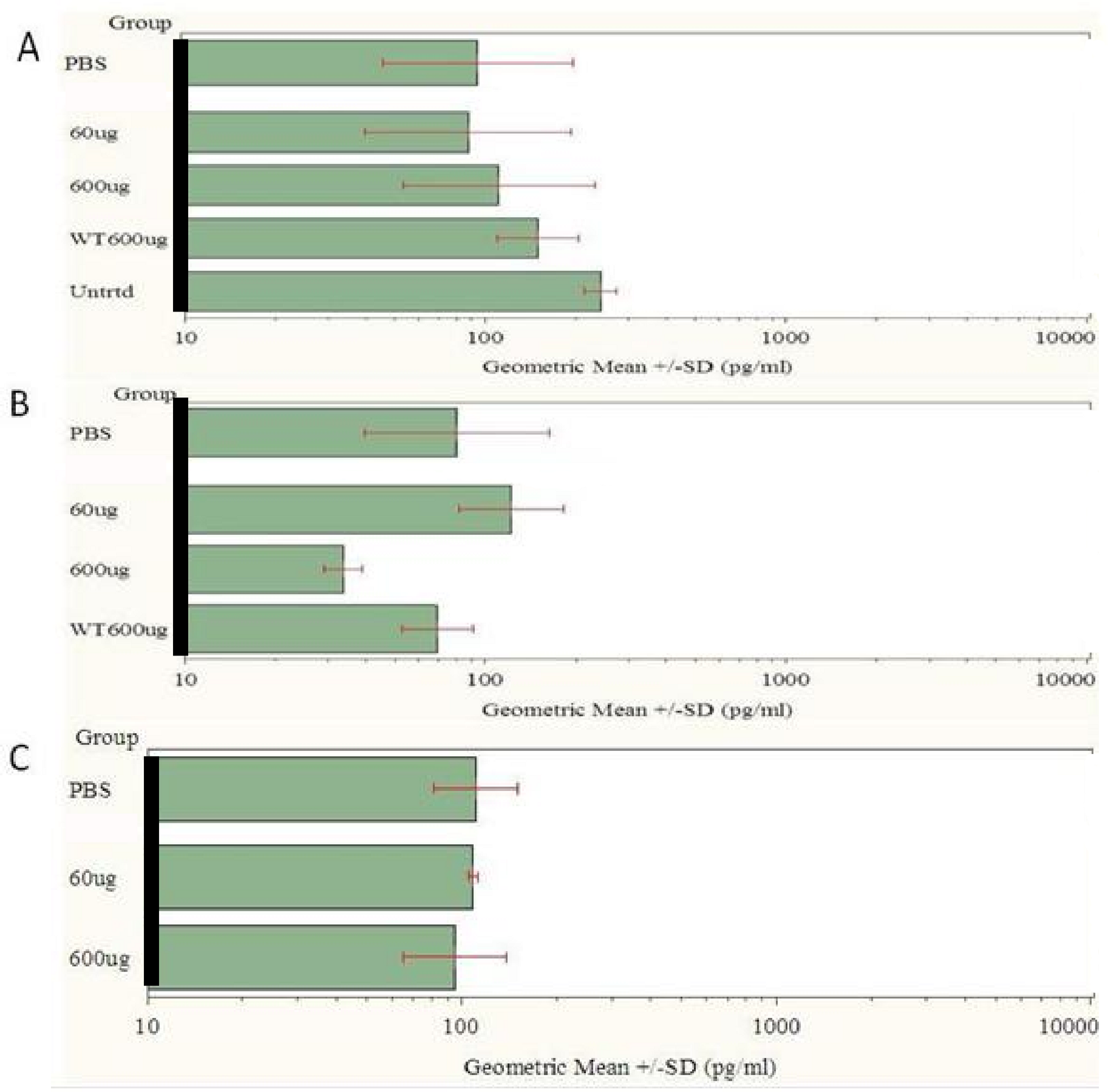

Supplement: Figure S3 — Free Aβ (1–40 & 1–42) levels in cfh−/− mouse plasma after therapeutic administration regime. Concentration of free Aβ (1–40 &1–42) in plasma samples are shown as geometric means with standard deviation at baseline (A), (n = 12/group) and after 4 weeks (B), (n = 4/group), and 12 weeks (C) of the therapeutic regime where final numbers were: PBS, vehicle n = 6; 6F6, 60 µg and 600 µg, n = 5. Labels are 6F6 dosed unless stated, Untrtd = untreated cfh−/− mice, WT600 µg = C57Bl/6 mice dosed with 6F6. Note a drop in plasma free Aβ (1–40 & 1–42) levels at a 600 µg dose of 6F6 at the 4 week time-point, which is close to statistical significance, over PBS controls p = 0.1416, (FDR adjusted), p = 0.0354 (non-adjusted), see Table S6. (TIF) [file pone.0065518.s003.tif]

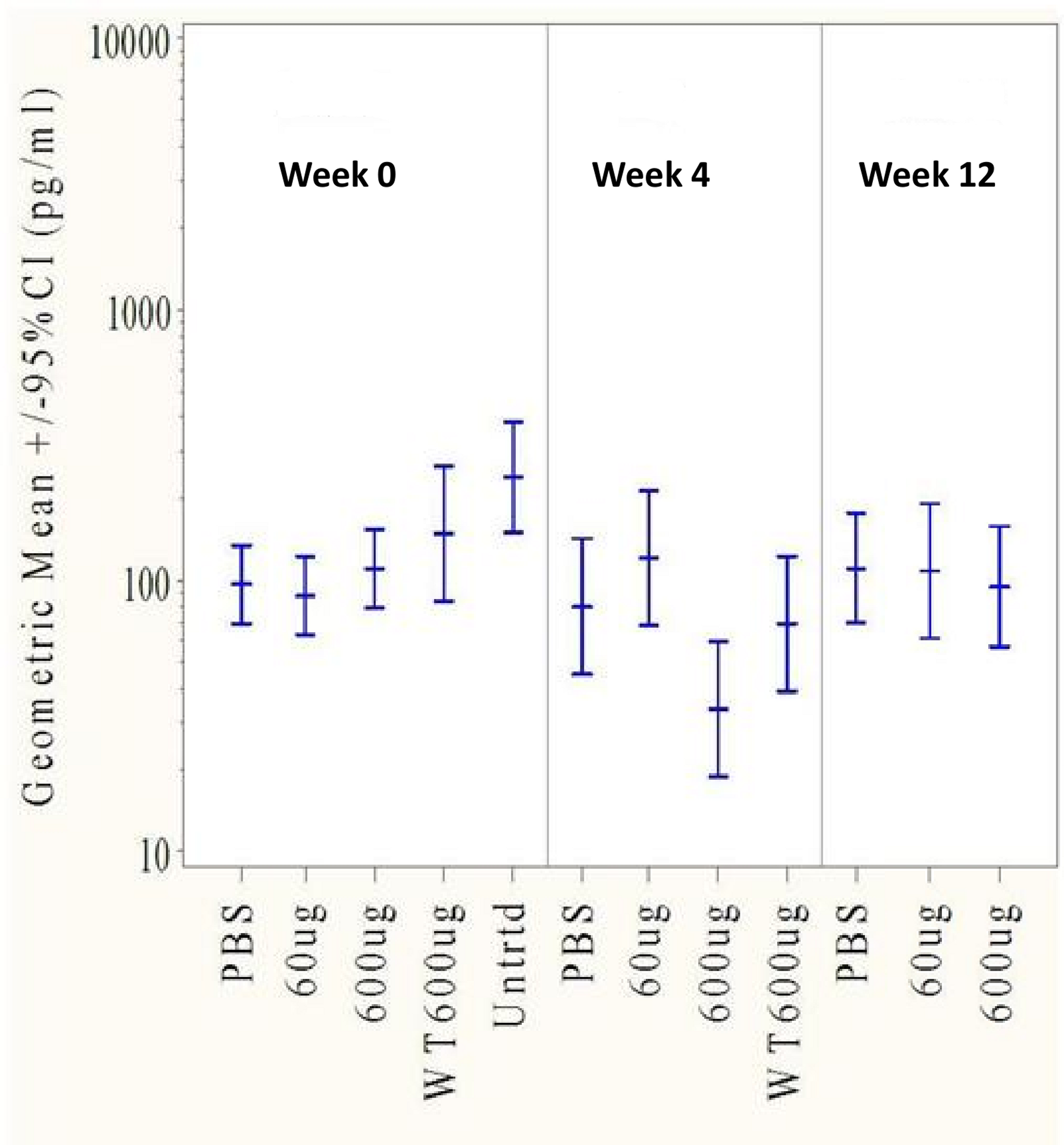

Supplement: Figure S4 — Geometric mean free Aβ (1–40 & 1–42) levels with 95% Confidence Intervals in cfh−/− mouse plasma after therapeutic administration regime. (TIF) [file pone.0065518.s004.tif]

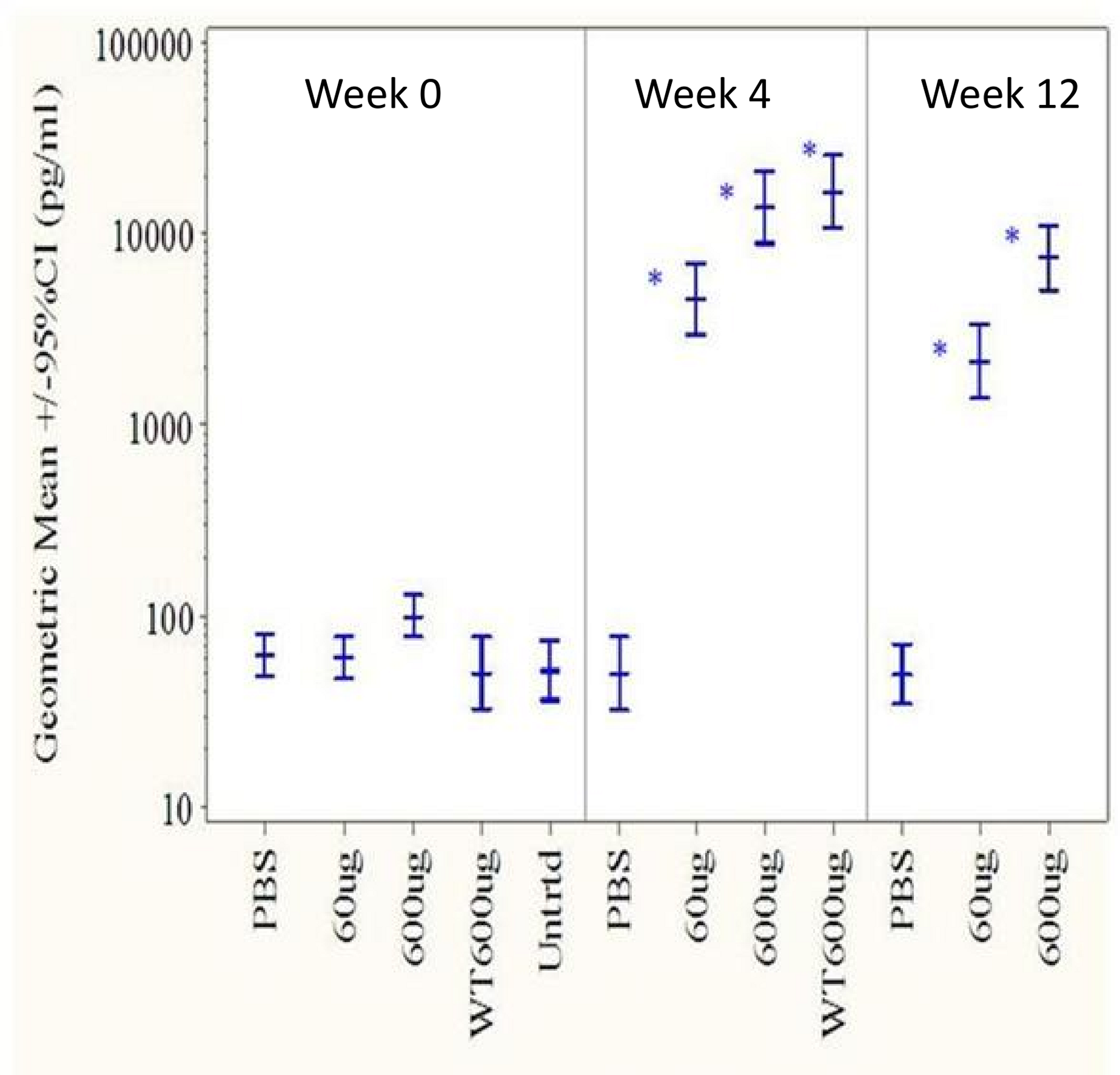

Supplement: Figure S5 — Geometric mean total Aβ 1–42 levels with 95% Confidence Intervals in cfh−/− mouse plasma after therapeutic administration regime. (TIF) [file pone.0065518.s005.tif]

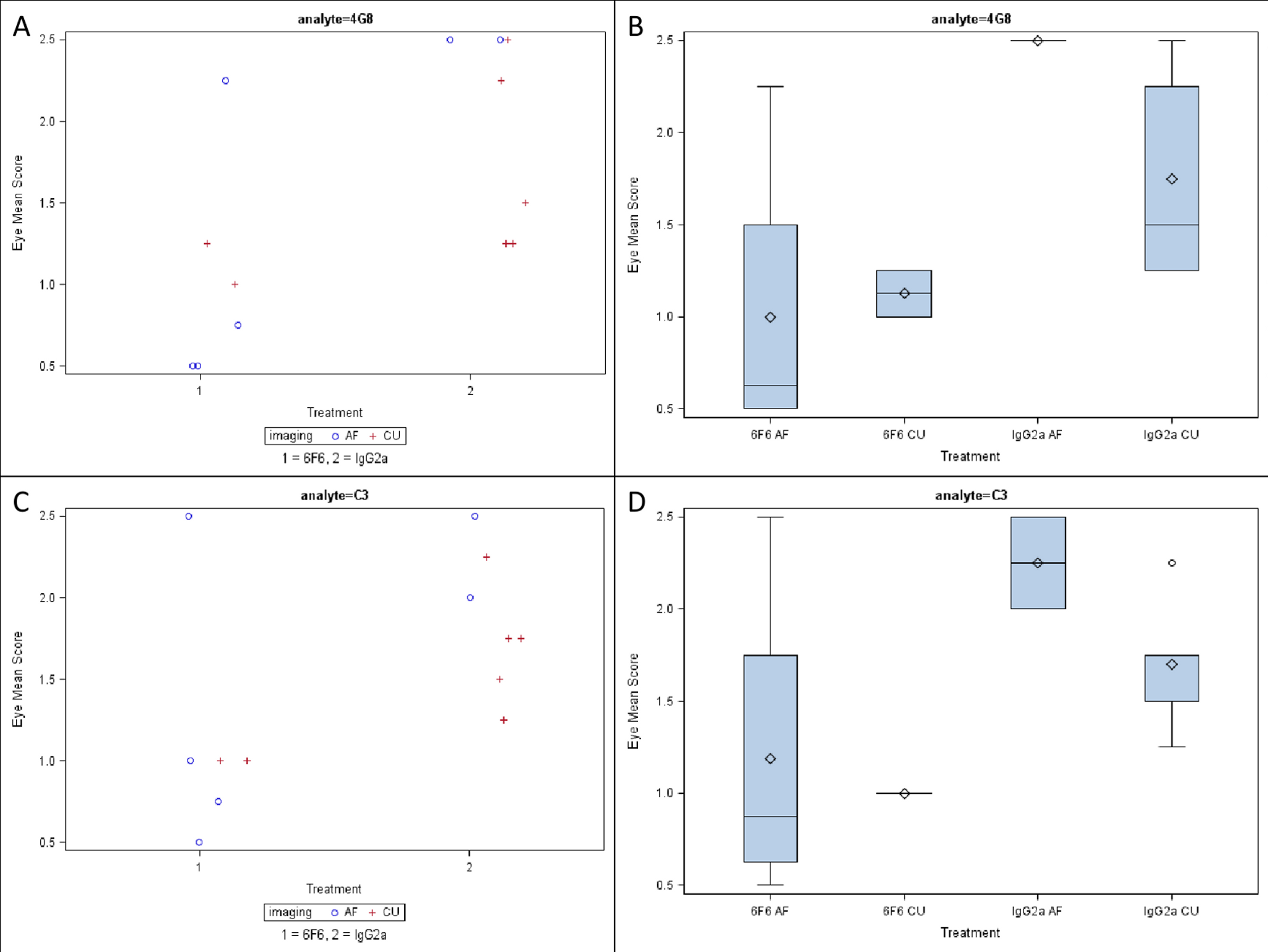

Supplement: Figure S6 — Comparison of relative spread of immunohistochemical scoring of the retinae of cfh−/− mice after treatment with 6F6 v IgG2A isotype control Ab. Data for geometric mean values of IHC scores for Amyloid β (4G8 = analyte): (A) scatter, (B) box & tail, and complement C3, detected with rabbit anti-rat polyclonal Ab to total C3, Hycult, HP80222, Table 3, (C3 = analyte): (C) scatter, (D) box & tail; were compared across the groups treated with either 6F6 or IgG2A and further sub-divided into sub-groups scored by standard autofluorescence, (AF, not further treated) and those additionally dosed with Curcumin, (CU). For the scatter plots: (A) & (C), a small constant greater than one was added to the data to highlight any overlapping points that might mask the true analysis of variability. For the 6F6 treated n = 7 eyes, (n = 4 AF, non curcumin treated, n = 3 curcumin treated) and for the IgG2A isotype control n = 6 eyes, (n = 2 AF, non curcumin treated, n = 4 curcumin treated). The IHC score ranges for the two subgroups +/− curcumin overlapped within each treatment group so it was considered a reasonable approach to pool the sub-groups for statistical analysis. (TIF) [file pone.0065518.s006.tif]

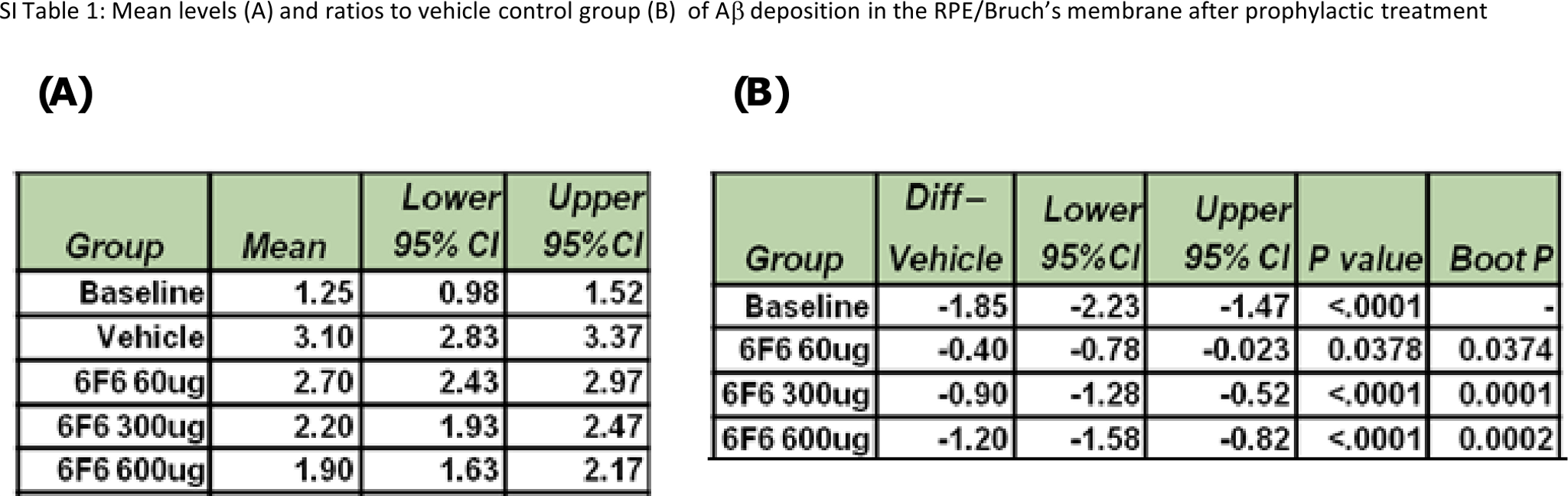

Supplement: Table S1 — Mean levels (A) and ratios to vehicle control group (B) of Aβ deposition in the RPE/Bruch’s membrane after prophylactic treatment. (TIF) [file pone.0065518.s007.tif]

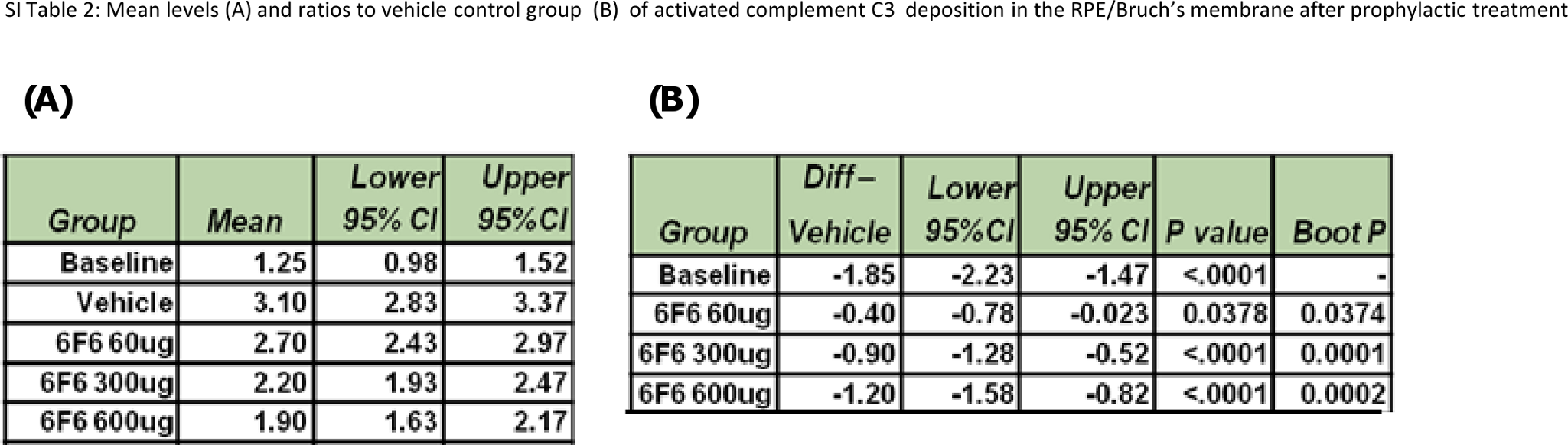

Supplement: Table S2 — Mean levels of activated complement C3 deposition (A) and ratios to vehicle controls (B) in the RPE/Bruch’s membrane after prophylactic treatment. (TIF) [file pone.0065518.s008.tif]

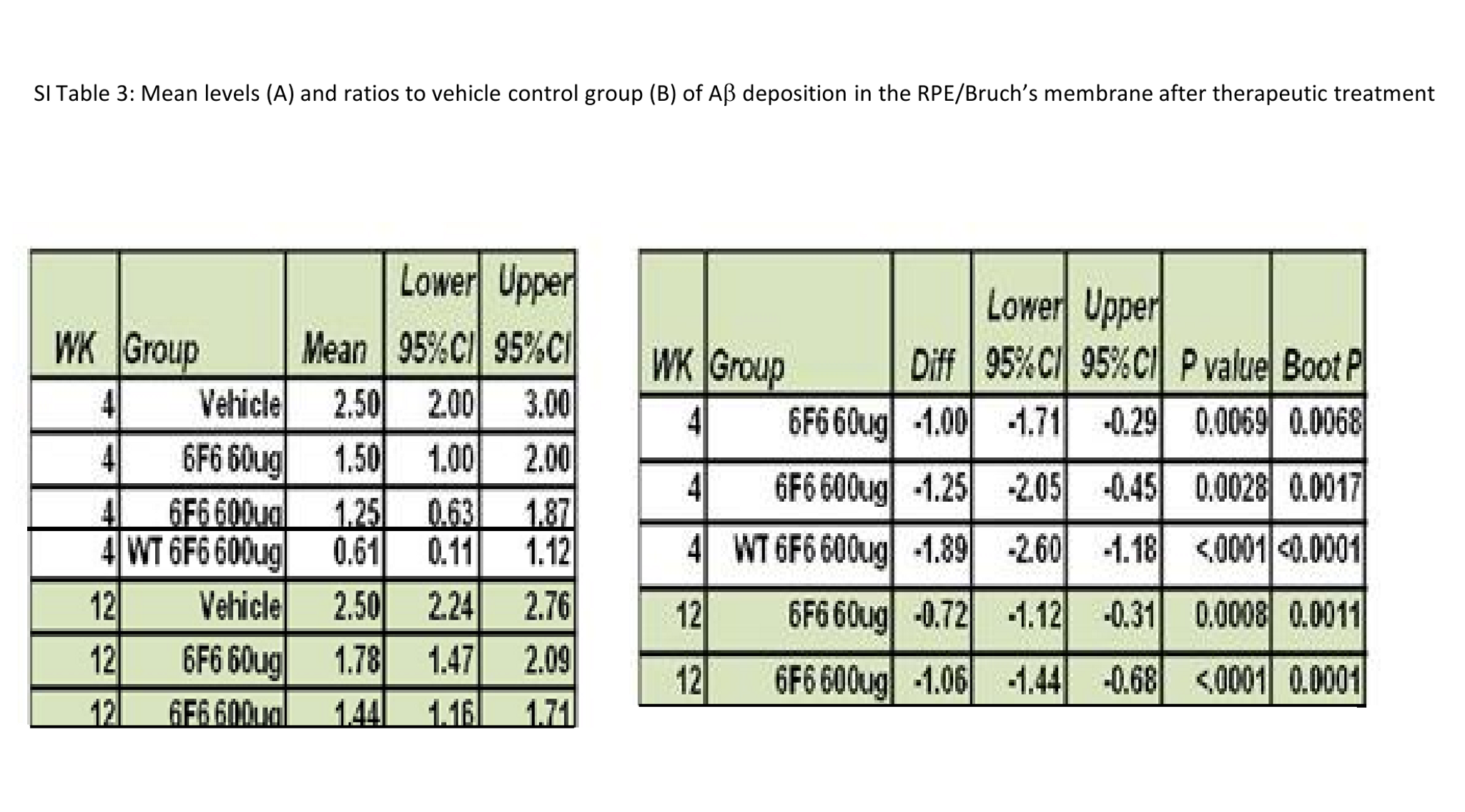

Supplement: Table S3 — Mean levels (A) and ratios to vehicle control group (B) of Aβ deposition in the RPE/Bruch’s membrane after therapeutic treatment. (TIF) [file pone.0065518.s009.tif]

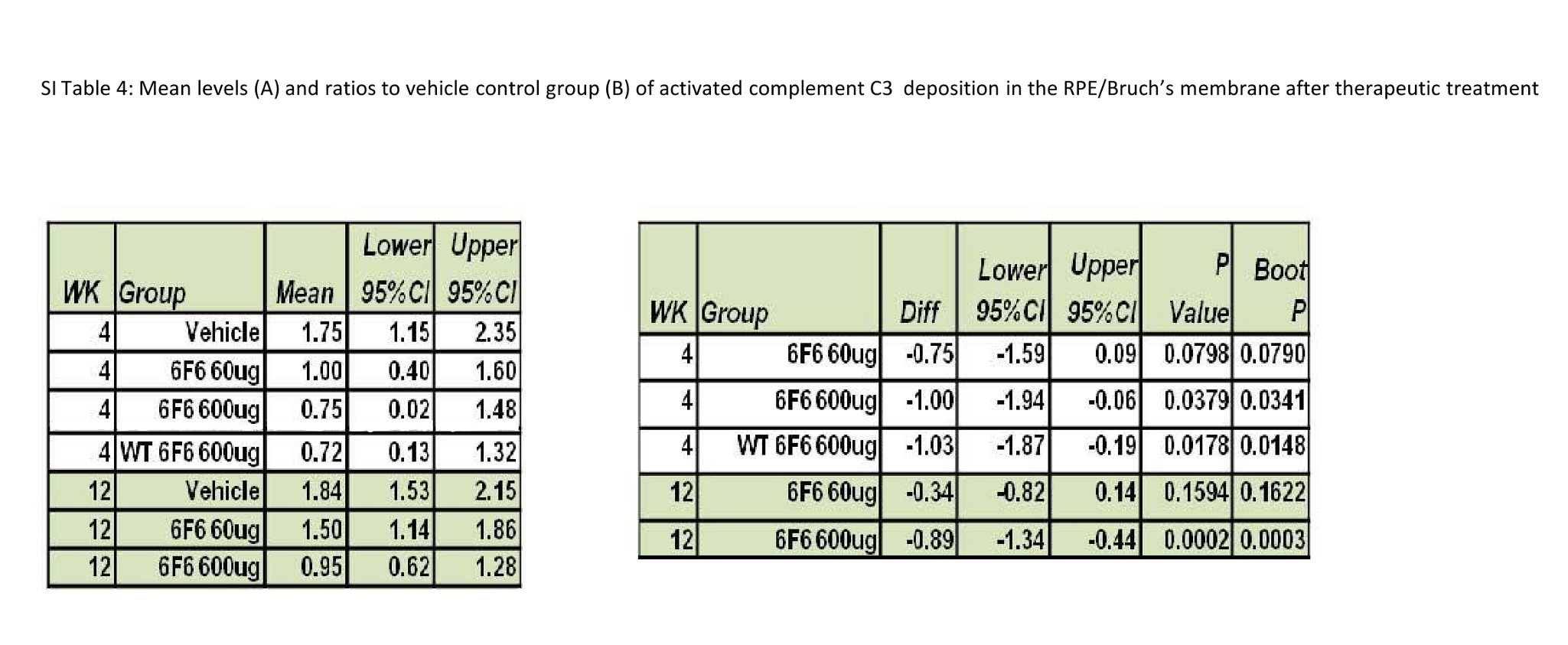

Supplement: Table S4 — Mean levels (A) and ratios to vehicle control group (B) of activated complement C3 deposition in the RPE/ Bruch’s membrane after therapeutic treatment. (TIF) [file pone.0065518.s010.tif]

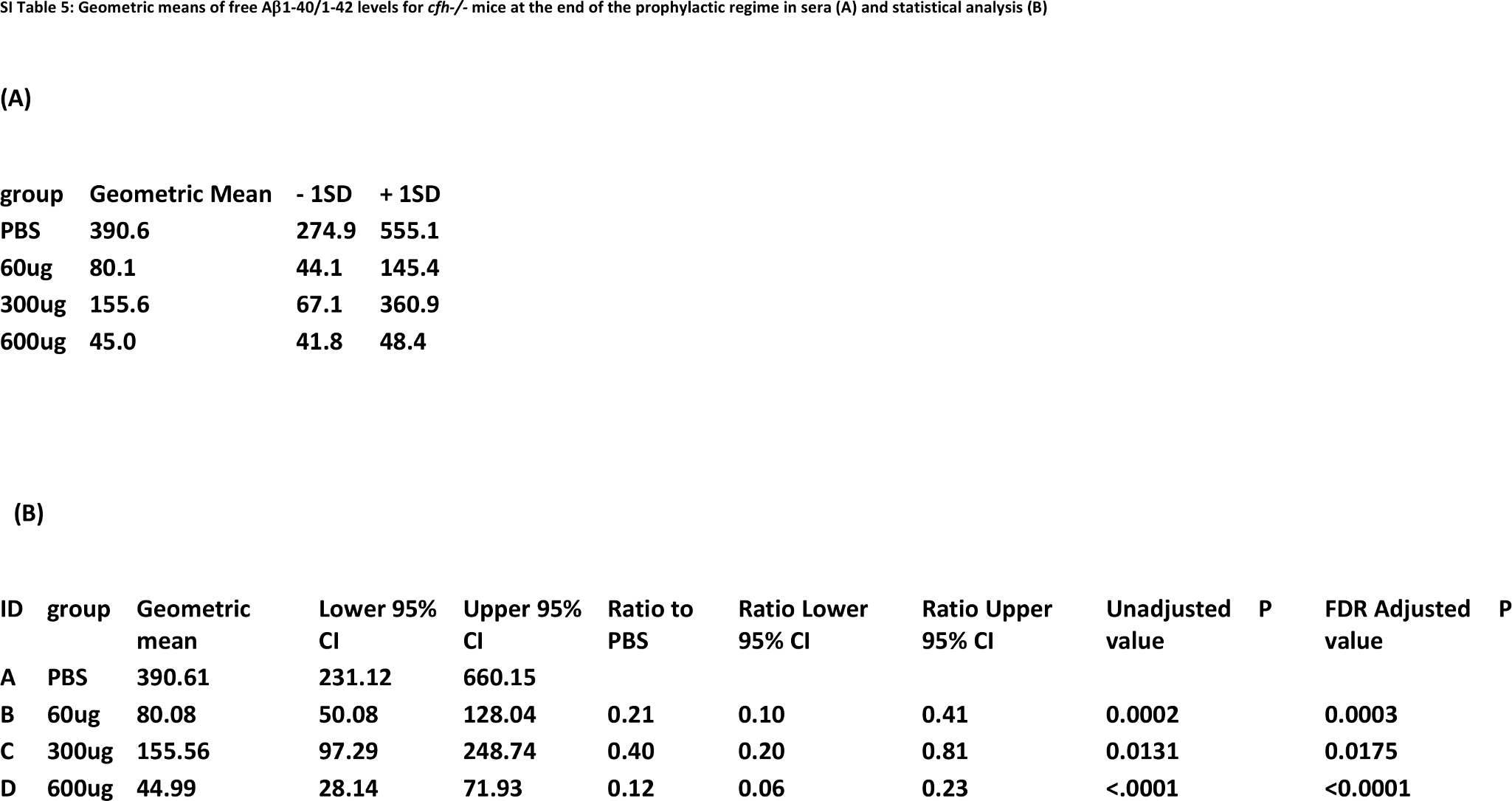

Supplement: Table S5 — Geometric means of free Aβ 1–40/1–42 levels for cfh−/− mice at the end of the prophylactic regime in sera (A) and statistical analysis (B). (TIF) [file pone.0065518.s011.tif]

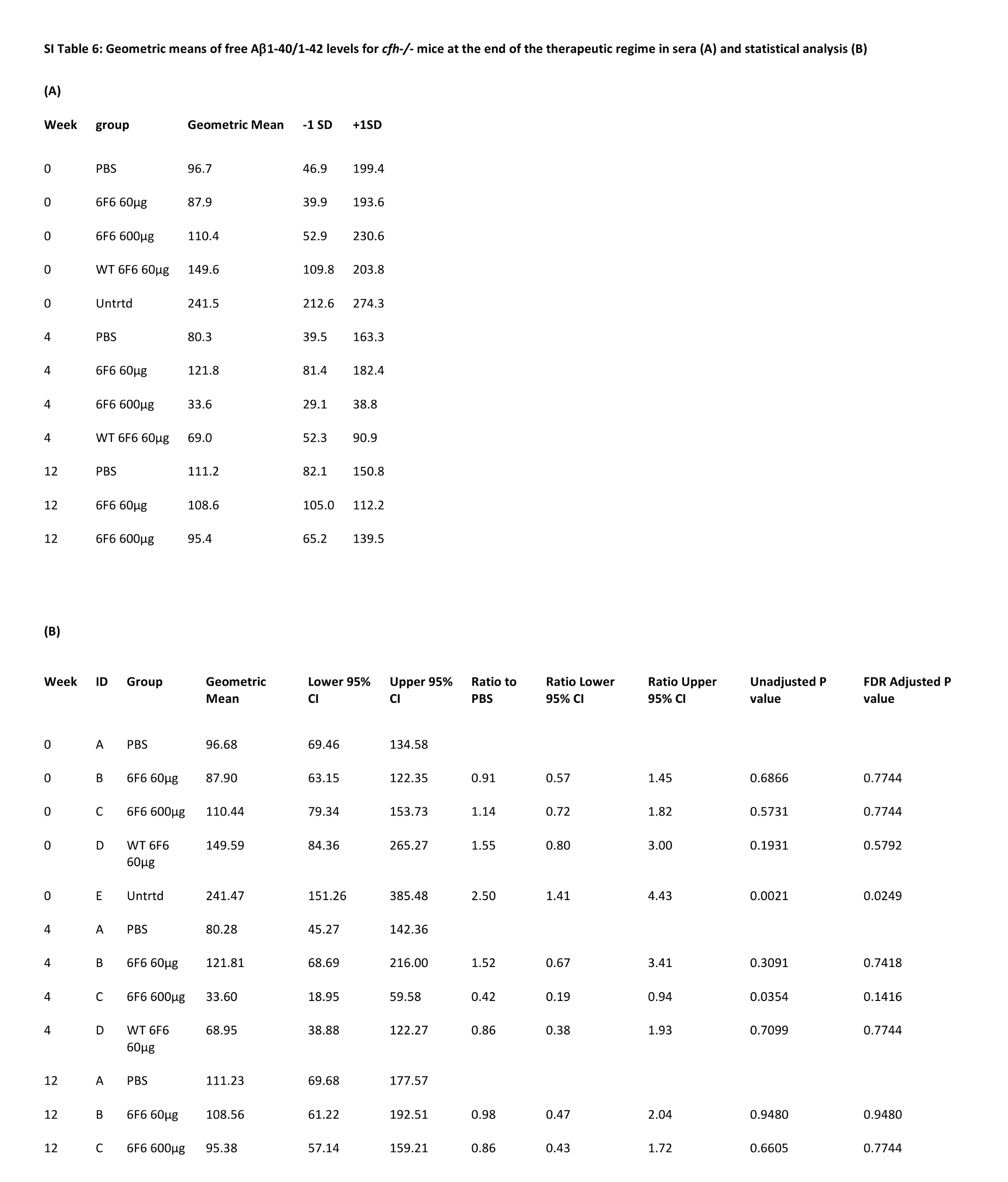

Supplement: Table S6 — Geometric means of free Aβ 1–40/1–42 levels for cfh−/− mice at the end of the therapeutic regime in plasma (A) and statistical analysis (B). (TIF) [file pone.0065518.s012.tif]

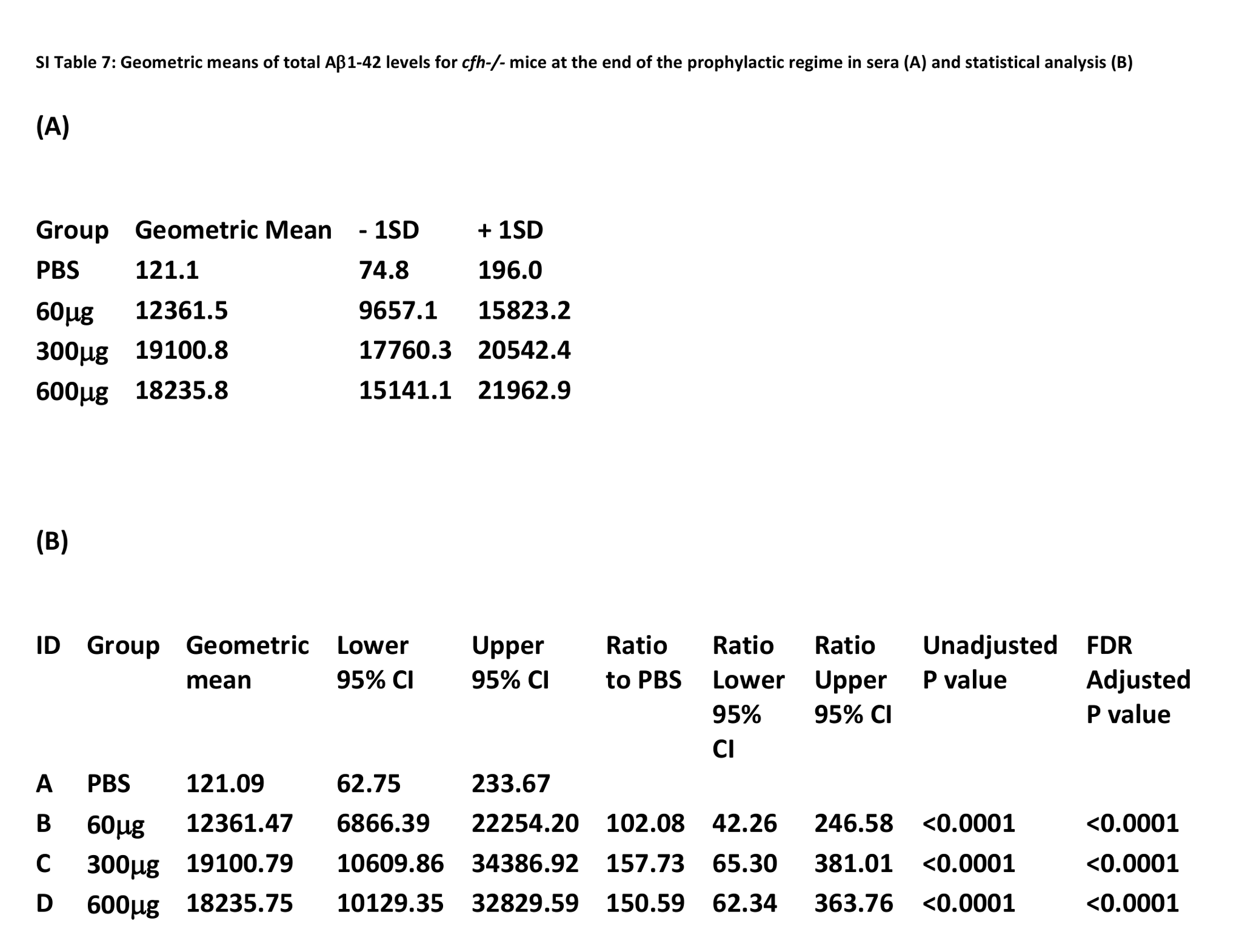

Supplement: Table S7 — Geometric means of total Aβ 1–42 levels for cfh−/− mice at the end of the prophylactic regime in sera (A) and statistical analysis (B). (TIF) [file pone.0065518.s013.tif]

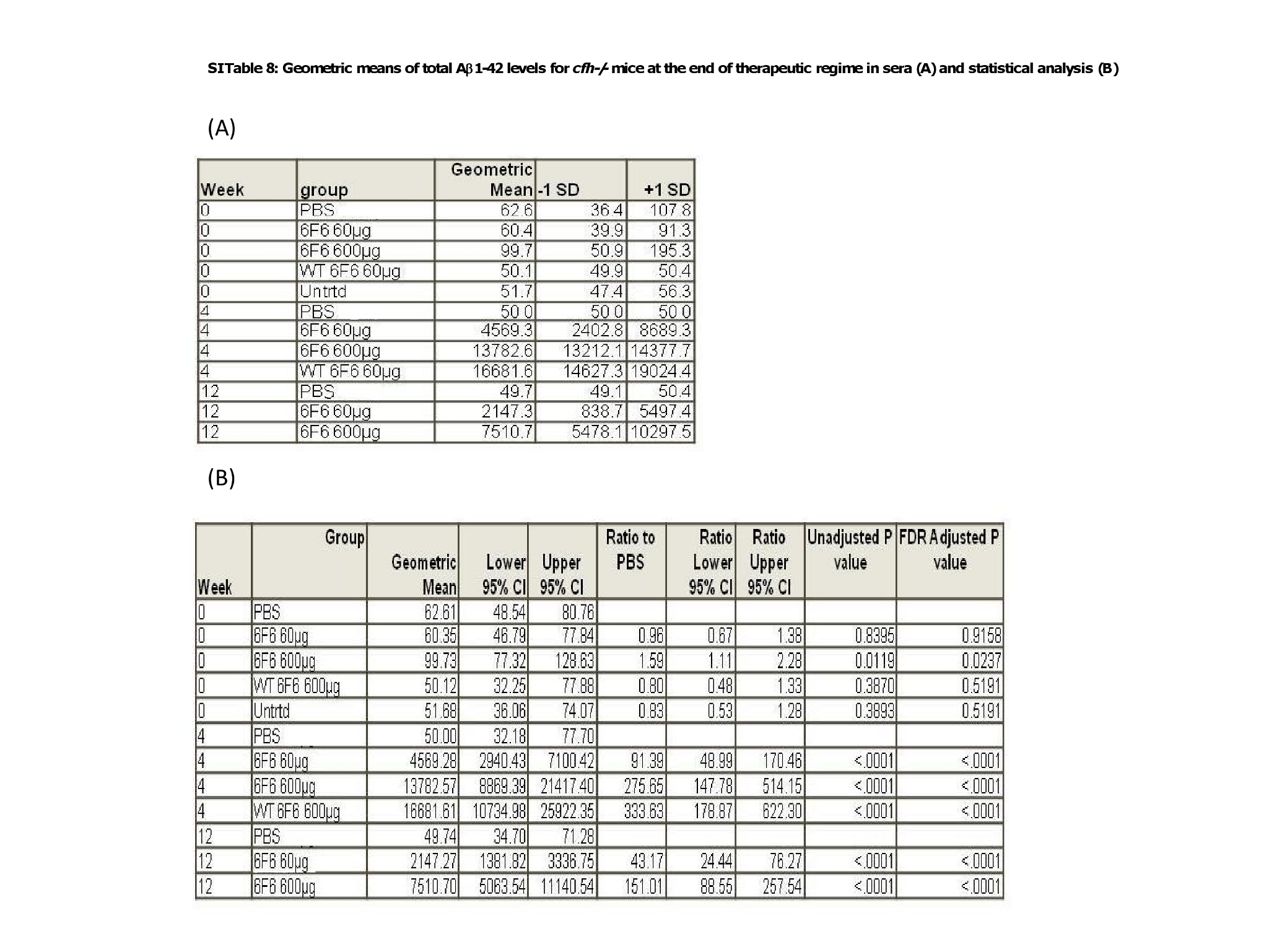

Supplement: Table S8 — Geometric means of total Aβ1–42 levels for cfh−/− mice at the end of the therapeutic regime in plasma (A) and statistical analysis (B). (TIF) [file pone.0065518.s014.tif]
