# Supplementary material for: Systemic Administration of Abeta mAb Reduces Retinal Deposition of Abeta and Activated Complement C3 in Age-Related Macular Degeneration Mouse Model
Source: PLoS One. 2013 Jun 14;8(6):e65518. doi: 10.1371/journal.pone.0065518 (PMC3682980; doi:10.1371/journal.pone.0065518)
Supplement: Methods S1 — Comparison of systemic administration of 6F6 to an IgG2A isotype control in cfh−/− mice using Curcumin as an imaging marker. (DOCX) [file pone.0065518.s015.docx]

**Methods: Supporting Information S1**

**Comparison of systemic administration of 6F6 to an IgG2A isotype control in *cfh-/-* mice using Curcumin as an imaging marker.**

Grading system for IHC analysis

IHC results in treated eyes for complement C3, (C3^+^) along Bruch’s membrane were graded according to degree of expression as follows:

1. Grade 0, <10% expression along Bruch’s membrane;
2. Grade 1, fragmental expression the length of 10–25% retinae;
3. Grade 2, segmental expression along 25–50% of Bruch’s membrane;
4. Grade 3, close to continues expression, or 50-100%,  the length of Bruch’s membrane;

For IHC studies of Aβ deposition (4G8^+^) along Bruch’s membrane and outer segments in sections were graded as follows:

- - - 1. Grade 0: No deposition along the Bruch’s membrane and limited expression on outer segment tips
      2. Grade 1, Fragmented deposition or ‹ 10% deposition along Bruch’s membrane and limited expression on outer segments
  1. Grade 2, Segmental deposition along 10-50% of Bruch’s membrane and extensive staining on outer segments

Grade 3, Close to continuous deposition, or 50-100%, along the length of Bruch’s membrane and solid expression on outer segment
